# The amygdala modulates prepulse inhibition of the auditory startle reflex through excitatory inputs to the caudal pontine reticular nucleus

**DOI:** 10.1186/s12915-021-01050-z

**Published:** 2021-06-03

**Authors:** Jose Carlos Cano, Wanyun Huang, Karine Fénelon

**Affiliations:** 1 Department of Biological Sciences, University of Texas at El Paso, 500 West University Avenue, El Paso, TX 79912 USA; 2 Biology Department, University of Massachusetts Amherst, Life Science Laboratories, 240 Thatcher Road, Amherst, MA 01002 USA

**Keywords:** Sensorimotor gating, Electrophysiology, Caudal pontine reticular nucleus, Amygdala, Optogenetics, Startle

## Abstract

**Background:**

Sensorimotor gating is a fundamental pre-attentive process that is defined as the inhibition of a motor response by a sensory event. Sensorimotor gating, commonly measured using the prepulse inhibition (PPI) of the auditory startle reflex task, is impaired in patients suffering from various neurological and psychiatric disorders. PPI deficits are a hallmark of schizophrenia, and they are often associated with attention and other cognitive impairments. Although the reversal of PPI deficits in animal models is widely used in pre-clinical research for antipsychotic drug screening, the neurotransmitter systems and synaptic mechanisms underlying PPI are still not resolved, even under physiological conditions. Recent evidence ruled out the longstanding hypothesis that PPI is mediated by midbrain cholinergic inputs to the caudal pontine reticular nucleus (PnC). Instead, glutamatergic, glycinergic, and GABAergic inhibitory mechanisms are now suggested to be crucial for PPI, at the PnC level. Since amygdalar dysfunctions alter PPI and are common to pathologies displaying sensorimotor gating deficits, the present study was designed to test that direct projections to the PnC originating from the amygdala contribute to PPI.

**Results:**

Using wild type and transgenic mice expressing eGFP under the control of the glycine transporter type 2 promoter (GlyT2-eGFP mice), we first employed tract-tracing, morphological reconstructions, and immunohistochemical analyses to demonstrate that the central nucleus of the amygdala (CeA) sends glutamatergic inputs lateroventrally to PnC neurons, including GlyT2^+^ cells. Then, we showed the contribution of the CeA-PnC excitatory synapses to PPI in vivo by demonstrating that optogenetic inhibition of this connection decreases PPI, and optogenetic activation induces partial PPI. Finally, in GlyT2-Cre mice, whole-cell recordings of GlyT2^+^ PnC neurons in vitro paired with optogenetic stimulation of CeA fibers, as well as photo-inhibition of GlyT2^+^ PnC neurons in vivo, allowed us to implicate GlyT2^+^ neurons in the PPI pathway.

**Conclusions:**

Our results uncover a feedforward inhibitory mechanism within the brainstem startle circuit by which amygdalar glutamatergic inputs and GlyT2^+^ PnC neurons contribute to PPI. We are providing new insights to the clinically relevant theoretical construct of PPI, which is disrupted in various neuropsychiatric and neurological diseases.

**Supplementary Information:**

The online version contains supplementary material available at 10.1186/s12915-021-01050-z.

## Background

Sensorimotor gating is the ability of sensory events to inhibit or “gate” motor outputs. Currently, prepulse inhibition (PPI) of the acoustic startle reflex task remains the gold standard operational measure of sensorimotor gating [1–3], used both in humans and various animal models [2, 4]. PPI is a paradigm in which a pre-stimulus of low intensity (“prepulse”) presented ~ 10–500 ms before a startle stimulus (“pulse”), attenuates the startle response [1–9]. Although PPI deficits are a hallmark of schizophrenia [1, 3], they also occur in other psychiatric disorders, such as obsessive-compulsive disorder [5, 6], Gilles de la Tourette syndrome [7], Huntington’s disease [10–12], and post-traumatic stress disorder [8] and other neurological disorders such as seizure disorders [13–15] and nocturnal enuresis [16]. PPI impairments are associated with, and often predictive of, cognitive disruptions and attentional problems. In fact, hallucinations, obsessions, and compulsions are thought to emerge as a result of a deficient gating system that prevents the brain from filtering out irrelevant sensory cues, actions, or thoughts [6]. At the present time, the reversal of PPI deficits in animal models and patients with schizophrenia is an efficient tool for antipsychotic drug screening [17, 18]. Despite this, the neuronal pathways and mechanisms underlying the PPI regulatory circuitry are still unresolved. Therefore, identifying the cell types and synaptic mechanisms involved in PPI is crucial to further our understanding of the neuronal underpinnings of sensorimotor gating. Knowledge of the PPI regulatory circuitry will also have clinical applications, expanding our insights of the pathophysiology of disorders with PPI deficits, towards developing and screening therapeutics for these disorders.

**Fig. 1:**
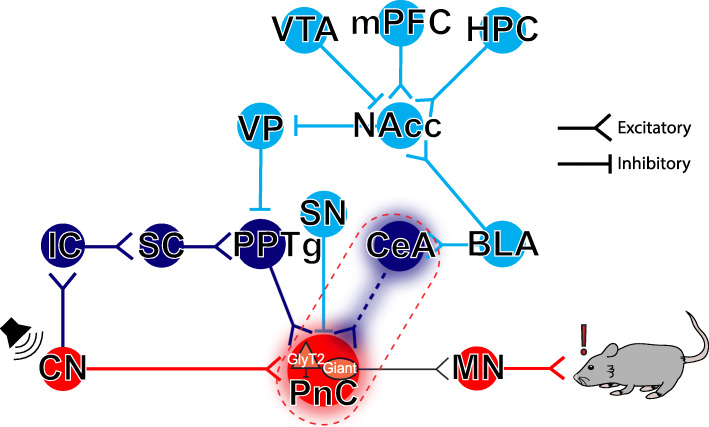
Neuronal circuits contributing to the acoustic startle response and PPI. The mammalian primary acoustic startle pathway (*red pathway*) consists of primary auditory neurons that activate cochlear root and cochlear nuclei (CN), which then relay the auditory information to the giant neurons of the caudal pontine reticular nucleus (PnC) in the brainstem. PnC giant neurons then directly activate cervical and spinal motor neurons (MNs). During PPI (*dark blue pathway*), acoustic prepulses are thought to inhibit startle via the activation of the inferior (IC), superior colliculi (SC), and the pedunculopontine tegmental nucleus (PPTg). The PPI pathway is also under the influence (*light blue pathway*) of midbrain and cortico-limbic structures including the basolateral amygdala (BLA), which activates the nucleus accumbens (NAcc) which in turn inhibits the ventral pallidum (VP). Together, these PPI structures form the cortico-striato-pallido-pontine (CSPP) network. Here, we propose that CeA-PnC excitatory synapses (*dotted dark blue pathway within the dotted red rectangle*) regulate PPI alongside the CSPP circuit. HPC: hippocampus; mPFC: medial prefrontal cortex; SN: substantia nigra; VTA: ventral tegmental area

The mammalian startle circuit is simple and consists of the cochlear nuclei, which activate giant neurons in the caudal pontine reticular nucleus (PnC) that, in turn, directly innervate cervical and spinal motor neurons (MNs) [4, 19, 20] (Fig. 1). Previous animal and human studies have shown that inhibition of this pathway by prepulses on the level of the PnC leads to PPI [1–9]. PPI by acoustic stimuli depends on the activation of midbrain structures including the inferior and the superior colliculi, as well as the pedunculopontine tegmental nucleus (PPTg) [21–24]. In addition, different cortical and subcortical areas within the corticostriatal-pallido-pontine (CSPP) circuit regulate PPI. In fact, manipulating the activity of the prefrontal cortex, thalamus, hippocampus, basolateral amygdala, nucleus accumbens, and dorsal striatum affects PPI [4, 25].

By receiving inputs from various sensory modalities (i.e., trigeminal and auditory) and directly activating spinal motor neurons, PnC giant neurons function as key sensorimotor relay neurons within the primary acoustic startle circuit. These PnC giant neurons receive cholinergic inputs from the PPTg, and general lesions of the PPTg were shown to disrupt PPI [22, 26]. Therefore, until recently, there was a consensus that PPI is mediated by cholinergic PPTg neurons inhibiting PnC giant neurons within the startle pathway. However, new optogenetic and chemogenetic rat studies clearly demonstrated that specific activation or inhibition of cholinergic PPTg neurons does not alter PPI [27–30]. Since the majority of neurons in the PPTg are non-cholinergic [31], it is currently suggested that PPI may be a function of GABAergic and/or glutamatergic cells in the PPTg and/or another structure directly projecting to the PnC. In fact, fish and rodent studies have described the crucial involvement of brainstem glutamatergic, GABAergic, and glycinergic inhibitory mechanisms in PPI [29, 30, 32].

As it is now clear that PPI does not depend on PPTg cholinergic inputs to the PnC, we aimed to identify what structure, other than the PPTg, projects directly onto PnC neurons and modifies PPI. One structure particularly relevant is the central nucleus of the amygdala (CeA), since various neurological disorders showing PPI deficits also show amygdalar abnormalities. In fact, the amygdala is a region that has received considerable attention in studies of the etiology of neuropsychiatric illnesses [33], and impairment of amygdala function disrupts PPI [34–38]. We hypothesized that the CeA-to-PnC connection provides an alternative PPI pathway, independent from the PPTg mechanisms. Our hypothesis is based on in vivo and in vitro rat studies that corroborate the important role of the CeA in modulating the startle pathway. The CeA receives inputs from the auditory cortex and the central thalamus [39, 40] and sends projections to the PnC [41, 42] making it a potential PPI substrate. More precisely, tract-tracing studies in rats showed that neurons in the rostral part of the medial subdivision of the CeA directly innervate PnC giant neurons at the core of the acoustic startle circuit [42, 43]. However, in contrast to PPI where startle magnitude is *decreased* by a non-startling prepulse, early behavioral studies focused on understanding how startle magnitude is *increased* during conditioned and unconditioned states of fear, as seen both in rats [44, 45] and in humans [46]. These studies, performed in rats, show that the acoustic startle response is enhanced by electrically stimulating the amygdalobasal complex, including the CeA [44] or by injecting NMDA bilaterally in the amygdaloid complex [43]. In vivo electrophysiological recordings also revealed that activating the CeA/amygdaloid complex yields excitatory post-synaptic potentials and enhances the acoustic responsiveness of PnC giant neurons in rodents [43, 47].

It is only recently that functional imaging studies and c-Fos expression data in rats have provided strong evidence that CeA neuronal activity is increased during PPI [48]. However, whether and how the CeA-to-PnC excitatory connection, specifically, contributes to PPI has never been tested. Moreover, downstream inhibitory PnC neuronal elements that could help reconcile the role of CeA excitatory projections in a functionally inhibitory pathway remain to be identified.

Interestingly, glycinergic fibers and interneurons expressing the glycine transporter type 2 (GlyT2) are closely apposed to the PnC giant neurons in rodents [49, 50]. Although the role and source of activation of these GlyT2^+^ neurons are unclear in the context of PPI, glycine neurotransmission has been shown to inhibit rat PnC giant neurons and contribute to PPI at the level of the startle-initiating Mauthner cells, within the goldfish auditory startle circuit [32, 51].

The present study was undertaken to test the hypothesis that glutamatergic CeA neurons contribute to PPI by sending inputs to the PnC, including GlyT2^+^ neurons in mice. Here we used tract-tracing, morphological reconstructions, and neurochemical analyses and examined synaptic properties of glutamatergic CeA inputs terminating primarily onto GlyT2^+^ PnC neurons, using transgenic GlyT2-eGFP and GlyT2-Cre mice. We validated our findings in vivo by specific optogenetic inhibition and activation of CeA-PnC glutamatergic synapses, as well as optogenetic inhibition of GlyT2^+^ PnC neurons during startle and PPI testing.

## Results

### CeA glutamatergic neurons send inputs to the PnC lateroventral region in mice

**Fig. 2:**
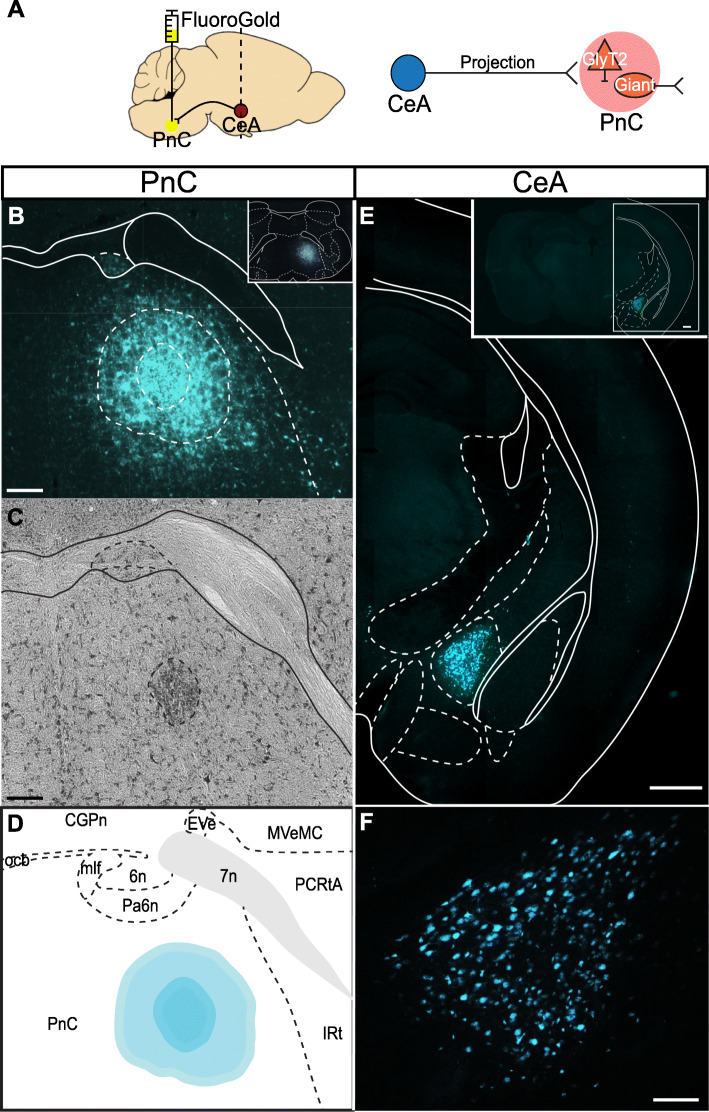
The CeA sends projections to the PnC. **a** *Left*, Sagittal representation of the mouse brain illustrating the Fluoro-Gold injection site in the PnC (yellow circle) and the retrograde labeling site in the CeA (red circle, dotted line). *Right*, Schematic of the hypothesis being tested. **b** Representative coronal PnC slice showing the extent of the Fluoro-Gold injection. The outer dotted circle indicates the fluorescent Fluoro-Gold injection halo. The inner dotted circle represents the center of the gliotic lesion, medial to the 7th cranial nerve and within the cytoarchitectural boundaries of the PnC. *Inset:* Representative image of the injection site in a coronal PnC slice, shown at lower magnification. **c** Representative Nissl-stained PnC section. The darker region surrounded by a dotted circular area indicates the gliotic lesion made by the Fluoro-Gold injection. **d** Representative image showing the Fluoro-Gold injection site in the PnC mapped to the Paxinos and Franklin Mouse Brain Atlas [52]. **e** Representative coronal section showing CeA neurons retrogradely labeled with Fluoro-Gold (cyan). **f** Higher magnification of the CeA neurons shown in **e** and retrogradely labeled with Fluoro-Gold. Representative of *N* = 4 mice. Scale bars: **b–d** 200 μm, **e** 500 μm, **f** 100 μm

As previously reported in rat tracing studies [42, 43], we first confirmed that the retrograde tracer Fluoro-Gold, injected unilaterally within the mouse PnC (Fig. 2a, b), labeled neurons in various brain regions. The cytoarchitectural analysis of the gliotic lesion [53] (Fig. 2d) showed that Fluoro-Gold was injected within the PnC, delineated by 7th nerve fibers. As expected, Fluoro-Gold labeled neurons in various regions including the caudate putamen, the intra-amygdaloid division of the bed nucleus of the stria terminalis, and the lateral hypothalamus. Fluoro-Gold also labeled neurons located in the mouse pedunculopontine tegmental area (PPTg) (Additional file 1: Figure S1; *N* = 4) and in the CeA (Fig. 2f; *N* = 4). Labeled CeA cell bodies clustered near the border of the dorsomedial portion of the anterior amygdalar complex, ipsilateral to the PnC injection site.

**Fig. 3:**
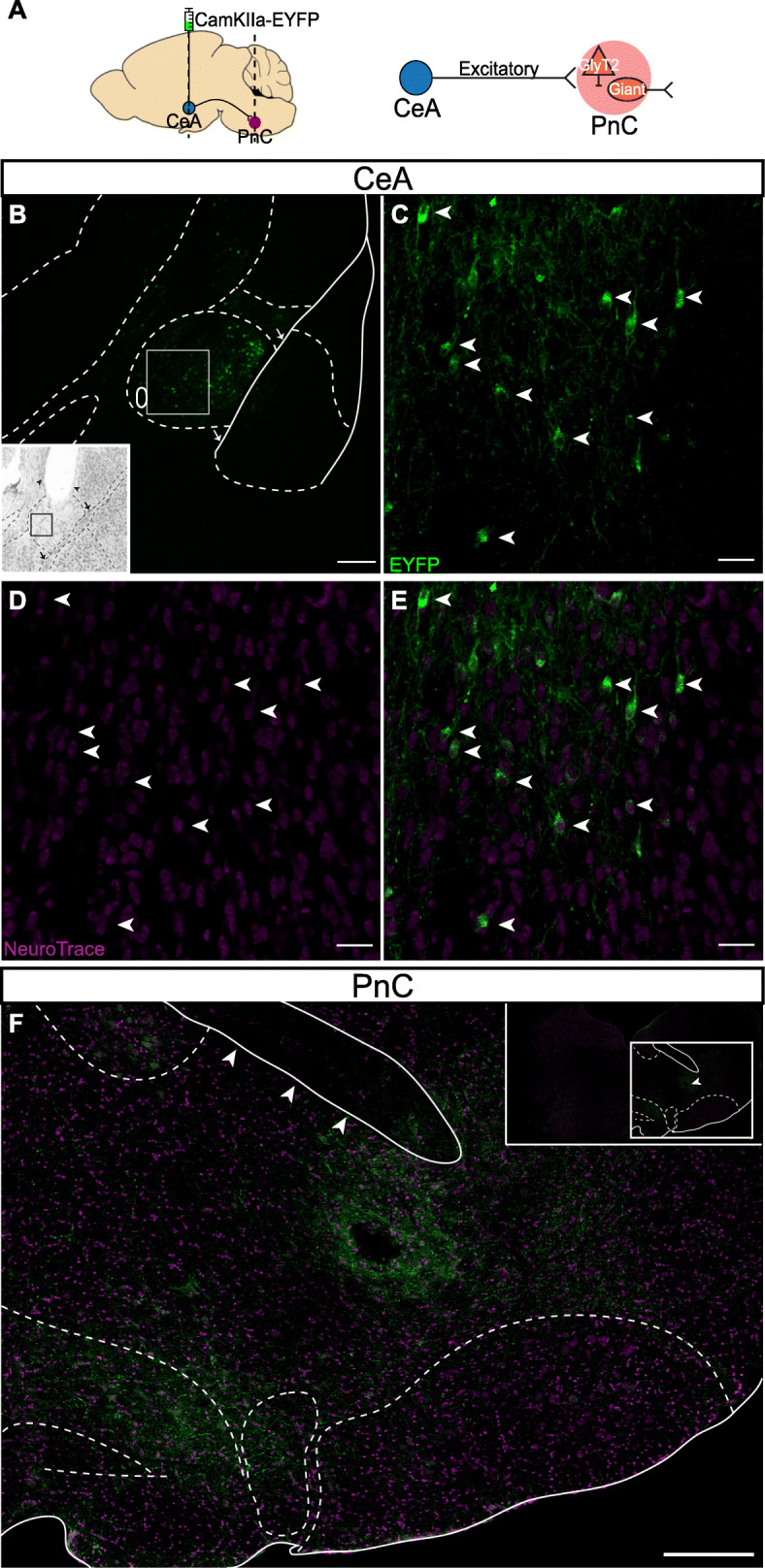
CeA glutamatergic projections course within the lateroventral portion of the PnC. **a** *Left*, Sagittal representation of the mouse brain illustrating the AAV-CamKIIα-eYFP injection site targeting CeA neurons (green circle) and CeA projection fibers terminating at the level of the PnC (red circle). The dotted line illustrates the PnC level at which coronal cut sections were obtained to visualize CamKIIα-eYFP^+^ axons originating from the CeA. *Right*, Schematic of the hypothesis being tested. **b** Representative CeA coronal sections showing eYFP^+^ fluorescence (green) and NeuroTrace^TM^ staining (magenta). The white rectangle shows the area imaged in panels **c**–**e**. *Inset*, Nissl stain image of the injection site in the CeA. Arrowheads represent the injection needle tract, dorsal to imaging site. The black square corresponds to the white square area on the fluorescence image. **c** White arrows indicate CeA cells positive for CamKIIα-eYFP (green). **d** NeuroTrace^TM^ stain (magenta) labels CeA cells bodies (white arrows). **e** White arrows indicate CeA cells positive for CamKIIα-eYFP and NeuroTrace^TM^. **f** Representative image of CamKIIα-eYFP^+^ CeA fibers (green) coursing within a PnC coronal section stained with NeuroTrace^TM^ (magenta). The three arrowheads indicate the 7th cranial nerve. *Inset*, lower magnification of the PnC coronal section with PnC delineated landmarks. The arrowhead indicate the location of the track left by the implanted optic fiber for PPI in vivo experiments. Representative of *N* = 4 mice. Scale bars: **c**, **e** 400 μm, **d**–**f** 50 μm

**Fig. 4:**
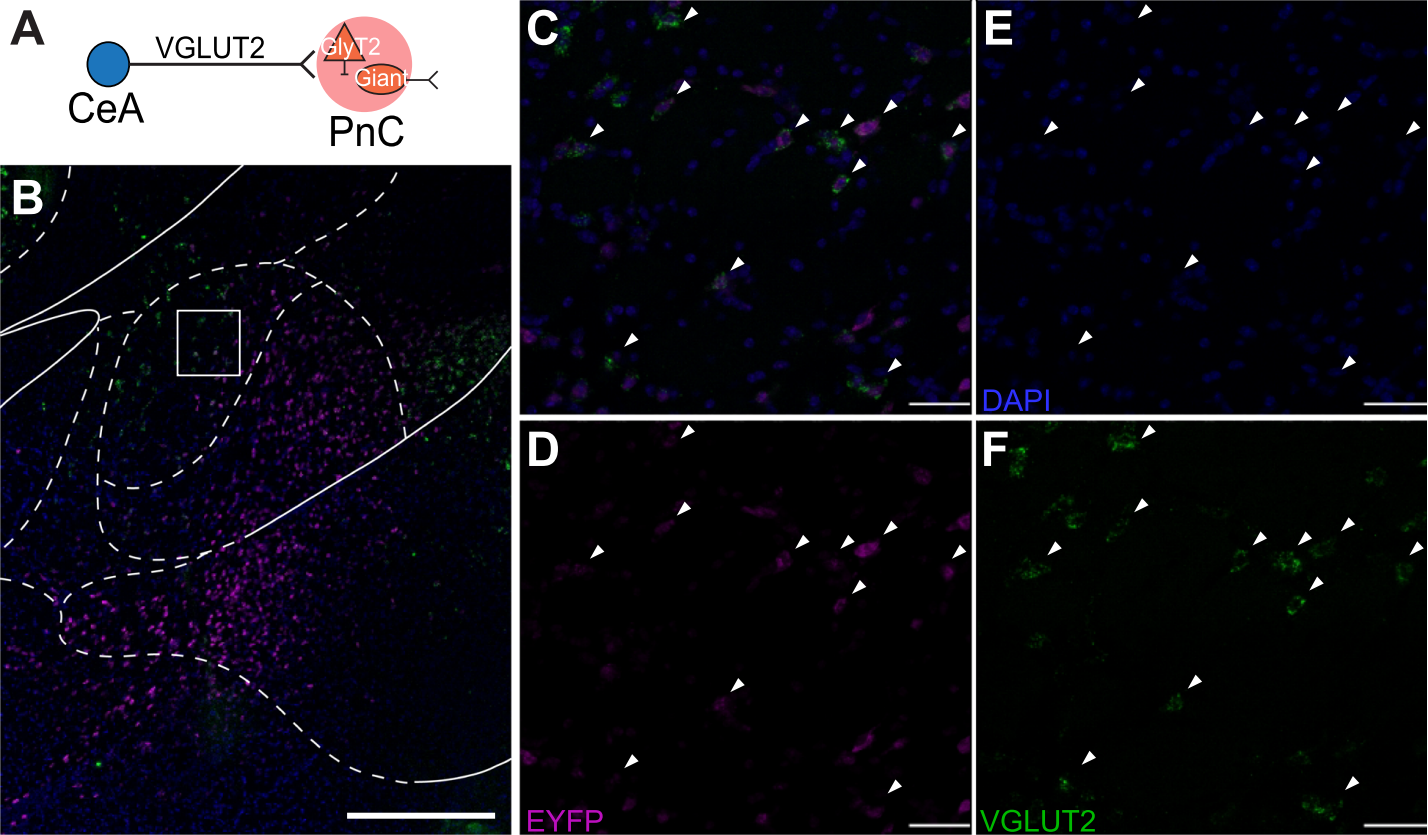
CeA neurons targeted with the AAV-CamKIIα-eYFP viral injection are VGLUT2^+^. **a** Schematic of the hypothesis being tested. **b** Representative image of a CeA coronal section at low magnification, hybridized with eYFP (magenta) and VGLUT2 (green) probes. White rectangle shows area imaged in panels **c**–**f**. **c**–**f** Arrowheads indicate CamKIIα-eYFP^+^ medial CeA neurons co-expressing VGLUT2 mRNA. Representative of *N* = 3 mice. Scale bars: **b** 500 μm, **c–f** 25 μm

The CeA comprises an array of distinct neuronal populations, including inhibitory neurons that have been classified according to different amygdala markers [54]. The CeA also contains excitatory neurons exhibiting VGLUT2 immunoreactivity, as shown in guinea pigs [55]. Since recent evidence suggests that midbrain glutamatergic inputs targeting the pontine startle circuit are important for sensory gating in zebrafish [29, 30], we next focused on how axons from CeA excitatory neurons course within the PnC (Fig. 3; *N* = 4). In mice injected with the viral vector AAV-CamKIIα-eYFP into the CeA (Fig. 3a), NeuroTrace^TM^ staining allowed us to confirm that the cell body of eYFP^+^ CeA neurons was efficiently targeted by the viral injection (Fig. 3b–e). Our results show that 11.29% (27 ± 13 somata) of CeA neurons labeled with Neurotrace^TM^ were eYFP^+^ (*N* = 4). Although it is often assumed that CamKIIα is specific to excitatory cells, some GABAergic projection neurons also express CamKIIα [56]. Therefore, we also identified the neurochemical nature of the CamKIIα-eYFP^+^ CeA neurons (Fig. 4a, b) using an in situ hybridization assay (RNAscope). We observed that eYFP^+^ CeA neurons express VGLUT2 (Fig. 4c–f, *N* = 3), and, as expected, these neurons were not co-labeled with a GABA antibody (Additional file 1: Figure S2). In fact, within the medial CeA, almost all (83% ± 8%) eYFP^+^ CeA neurons expressed VGLUT2. Finally, we observed that eYFP^+^ CeA axons course predominantly ipsilaterally in PnC sections and are localized in the lateroventral portion of the PnC (Fig. 3f).

### Optogenetic inhibition of CeA-PnC excitatory synapses decreases PPI

**Fig. 5:**
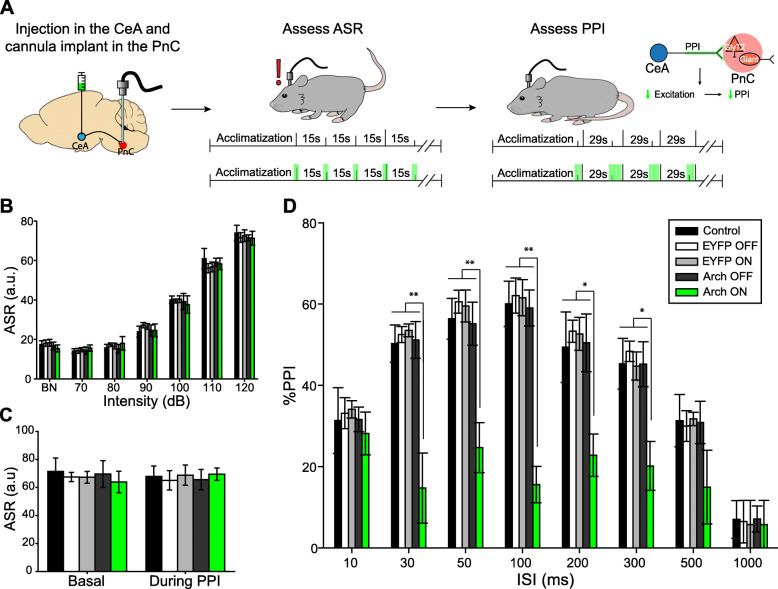
Silencing CeA-PnC excitatory projections during acoustic prepulses and ISIs decreases PPI. **a** Schematic of acoustic startle reflex and PPI protocols performed using non-injected WT control mice, mice injected with eYFP only (light ON or OFF), and mice injected with Archaerhodopsin (Arch3.0; light ON or OFF). The rightmost schematic represents the hypothesis being tested. **b** Graph showing no significant effect of green light presented prior to and during 70–120 dB acoustic pulses on basal startle amplitude among animal groups [mouse group: (F_(1,11)_ = 1.417, *p* = 0.268); light: (F_(1)_ = 0.00155, *p* = 0.969); sound intensity × light interaction: (F_(1,6)_ = 0.206, *p* = 0.974)]. **c** Graph showing no significant main effect of light during 120 dB pulses presented before (basal) vs. randomly during the PPI task, on mean baseline startle amplitude among animal groups (F_(1)_ = 3.124, *p* = 0.105). **d** Graph showing that green light paired with acoustic prepulses and ISIs significantly decreased PPI only in mice injected with Arch3.0, at ISIs between 30 and 300 ms. We found a significant effect of ISI (F_(1,7)_ = 24.863, *p* < 0.001), light: (F_(1)_ = 10.201, *p* = 0.009), and light × ISI interaction: (F_(1,7)_ = 4.057, *p* < 0.001) on PPI (Two-way RM ANOVA). *N* = 8 mice per group. Data are represented as mean ± SEM. *p < 0.05, **p < 0.01

Following our histological analyses, we assessed the potential role of the CeA-PnC excitatory connection during PPI, in vivo. We first aimed to determine if silencing amygdala neurons sending inputs to the PnC would alter baseline startle in the absence of a prepulse. To silence CeA-PnC excitatory synapses in WT mice, we transduced CeA excitatory cells with Archaerhodopsin-3 (Arch3.0) by injecting the optogenetic viral vector rAAVDJ/CamKIIa-eArch3.0-eYFP. We tested non-injected mice (control) and mice injected with the control rAAVDJ/CamKIIa-eYFP for comparison. Following the unilateral intracranial injection, an optic fiber was implanted at the level of the PnC to photo-inhibit CeA fibers/terminals expressing Arch3.0 (Fig. 5a, left).

To test our hypothesis that silencing CeA-PnC excitatory synapses does not alter baseline startle elicited by a pulse-alone stimulation (“pulse”), we photo-inhibited CeA-PnC excitatory synapses with green light prior to and concurrent with the acoustic stimulation at increasing sound levels, and then, we measured the startle response as a readout (Fig. 5a, middle). In all mice, sound intensities of and beyond 90 dB led to a measurable acoustic startle reflex, characterized by a whole-body flexor muscle contraction [2, 19, 20]. We found no differences when we compared the startle amplitude obtained with and without photo-inhibition of CeA-PnC excitatory synapses from eArch3.0-expressing animals to animals injected with the control virus and non-injected controls (Fig. 5b). These results, which were replicated using yellow light in mice injected with Halorhodopsin (pAAV DJ-CamKIIa-NpHR3.0-eYFP; Additional file 1: Figure S3A-B), confirm that inhibiting CeA-PnC excitatory synapses prior to and during a startle stimulation does not alter baseline startle. Our observations are consistent with previous rat studies demonstrating that lesions of the CeA do not block the acoustic startle response itself [57].

We then tested whether silencing CeA-PnC excitatory synapses alters PPI. To do so, the photo-inhibition started with prepulse onset and lasted until the end of the interstimulus intervals (ISIs) between prepulse and pulse (Fig. 5a, right) [26–29]. Although photo-inhibition had no impact on pulse-alone stimulations interspersed with PPI trials (Fig. 5c), photo-inhibition of CeA-PnC excitatory connection during the prepulse significantly decreased PPI by 25–43% at ISI between 30–300 ms in eArch3.0-expressing animals, but not in the control animals (Fig. 5d). Similarly, in mice injected with Halorhodopsin, photo-inhibition significantly decreased PPI by 16–29% when the prepulse was presented 50–300 ms before the startling pulse (Additional file 1: Figure S3C), without altering startle magnitude in pulse-alone trials (Additional file 1: Figure S3A-B). Since silencing the CeA-PnC excitatory connection during the prepulse and the subsequent ISI led to a decrease in PPI, these results support our hypothesis that CeA excitatory neurons regulate part of the behavioral PPI.

**Fig. 6:**
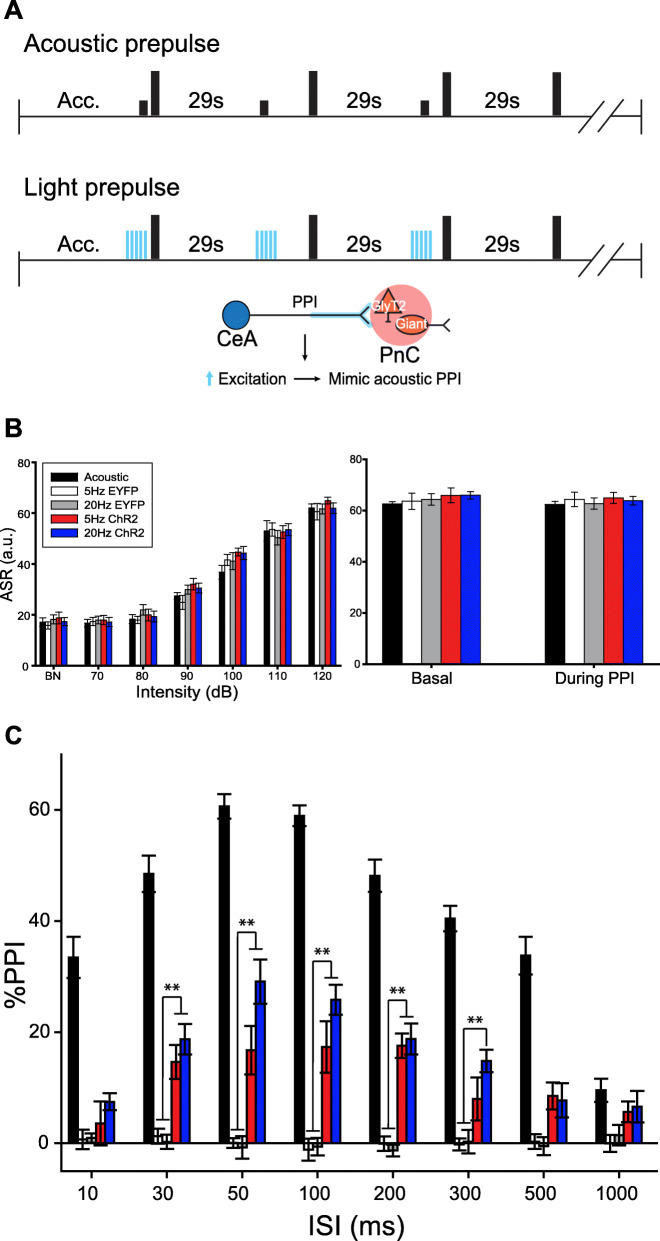
Photo-stimulation of CeA-PnC excitatory synapses induces PPI. **a** Representation of the PPI protocols performed using acoustic prepulses (*top*) or blue light prepulses (*middle*) in WT mice injected with eYFP only and mice injected with ChR2. The *bottom* schematic represents the hypothesis being tested. **b** In all mice, control (acoustic) ASR was assessed using acoustic pulse-alone startling stimulations (black bars). In addition, in WT mice injected with ChR2-eYFP, pulse-alone stimulations were paired with optogenetic stimulation trains of CeA-PnC glutamatergic synapses at 5 Hz (red bars) and 20 Hz (dark blue bars). Similarly, mice injected with the control vector AAV-eYFP were used to test possible blue light-induced heat effects at 5 Hz (white bars) and 20 Hz (gray bars) paired with pulse-alone stimulations. *Left*, Graph showing no significant main effect of blue light photo-stimulation paired with acoustic pulse-alone stimulations (70–120 dB) on mean basal startle amplitude, among animal groups. There was no effect of viral vector type (F_(1,4)_ = 2.096, *p* = 0.082) or viral vector × sound intensity interaction (F_(1,24)_ = 0.578, *p* = 0.944). *Right*, Graph showing no significant main effect of blue light photo-stimulation paired with 120-dB pulses presented during the PPI task, on mean baseline startle amplitude among animal groups. There was no effect of viral vector type (F_(1,4)_ = 1.250, *p* = 0.298) or viral vector × sound intensity interaction (F_(1,4)_ = 0.109, *p* = 0.979). **c** In all mice, control (acoustic) PPI was assessed using acoustic prepulses (black bars). In subsequent trials, photo-stimulation of CeA-PnC glutamatergic synapses at 5 Hz and 20 Hz replaced the prepulses in mice injected with the control AAV-eYFP vector (white and grey bars) and in mice injected with ChR2-eYFP (red and dark blue bars). The graph shows that only in mice injected with ChR2, optogenetic stimulation used as prepulses elicited PPI values 18–41% of acoustic prepulse, at ISI between 10 and 500 ms. (F_(1,14)_ = 6.152, *p* < 0.001). *N* = 8 mice per group. Data are represented as mean ± SEM. *p < 0.05, **p < 0.01

Next, we tested whether photo-activation of CeA-PnC excitatory synapses prior to a pulse-alone stimulation could mimic the effect of an acoustic prepulse and lead to PPI (Fig. 6a). WT mice injected with the control AAV eYFP vector were used to test for a possible blue light-induced heat effect. These mice were compared to WT mice injected with the optogenetic AAV vector rAAVDJ-CamKIIα-ChR2 in the CeA. In all mice, CeA-PnC excitatory synapses were photo-activated with short trains of blue light at 5 Hz and 20 Hz, concurrent with a startling pulse-alone stimulation. As shown in Fig. 6b, photo-activation of CeA-PnC excitatory synapses concurrent with an acoustic pulse-alone stimulation (Fig. 6b, *left*) or startling pulses interspersed with PPI trials (Fig. 6b, *right*) did not affect baseline startle. However, in all mice, CeA-PnC excitatory synapses were also photo-activated shortly prior to a startling pulse, at intervals used for acoustic PPI. Photo-activation at 5 Hz or 20 Hz prior to a startling stimulation produced a PPI effect across different ISIs (between photo-activation and acoustic pulses) only in WT mice injected with ChR2 (Fig. 6c). Interestingly, the 20 Hz photo-activation tended to be more efficient than the 5 Hz photo-stimulation train, yielding PPI values 18–41% of PPI elicited by the acoustic prepulses (all mice combined). Overall, the light-induced PPI effect was smaller than PPI induced by an acoustic prepulse, which resulted in a 35–60% PPI. Importantly, our results confirm that CeA excitatory neurons sending inputs to the PnC can inhibit startle at intervals relevant for PPI.

Finally, we tested whether photo-stimulation of CeA-PnC excitatory synapses (at 20 Hz) enhances the effect of the acoustic prepulse and potentiates PPI. Therefore, we paired the photo-stimulation with pulse-alone stimulations or with acoustic prepulses and ISIs during PPI trials, using mice injected with AAV-CamKIIα-ChR2-eYFP (Additional file 1: Figure S4; *N* = 6). Photo-stimulation did not alter baseline startle responses (Additional file 1: Figure S4A; *p* > 0.05) or PPI (Additional file 1: Figure S4B; *p* > 0.05). Based on our results, we conclude that CeA-PnC excitatory synapses are maximally activated following acoustic prepulses in vivo and that additional photo-stimulation of CeA-PnC excitatory inputs during acoustic prepulses and ISIs does not further enhance PPI.

### CeA glutamatergic neurons send inputs to PnC inhibitory neurons

**Fig. 7:**
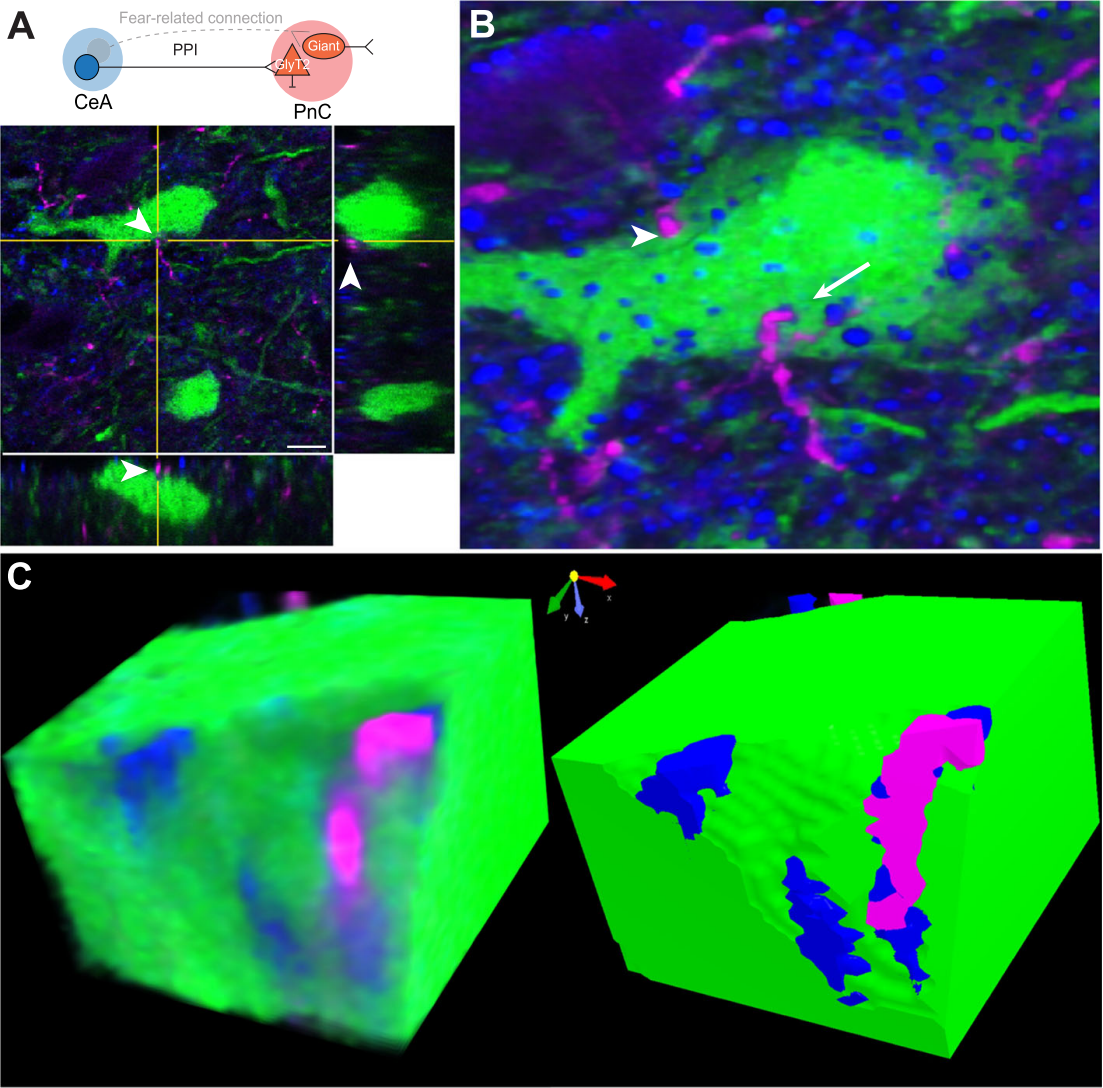
CamKIIα^+^ CeA fibers closely apposed to GlyT2^+^ PnC neurons. **a** *Top*, Schematic of the hypothesis being tested. *Bottom*, Orthogonal view of a close apposition between CamKIIα-mCherry^+^ CeA excitatory fibers (magenta) and the soma of a GlyT2^+^ PnC neuron (green) indicated by the arrowhead in all three views. **b** Three-dimensional reconstruction of putative synaptic contacts between CamKIIα-mCherry^+^ CeA fibers (magenta) and GlyT2-eGFP^+^ neurons expressing PSD-95 (blue; arrow). Few putative synaptic appositions did not show PSD-95 staining (arrowheads). **c** Volume rendering and angular sectioning of the PSD-95^+^ putative synaptic contact shown in **b**. Representative of *N* = 6 mice. Scale bar in **a** is 50 μm

We next aimed to reconcile the glutamatergic nature of CeA-PnC inputs and their role in a functionally inhibitory pathway. Since glycinergic neurons are found in the PnC and in close proximity to PnC giant neurons, we hypothesized that CeA excitatory inputs activate glycinergic PnC neurons, providing an inhibitory component to the PPI phenomenon. To test our hypothesis, we injected the AAV viral vector AAVDJ-CamKIIα-mCherry into the CeA of transgenic mice expressing eGFP under the control of the glycine transporter 2 (GlyT2) promoter [50]. Orthogonal imaging and morphological reconstruction analyses revealed close putative synaptic appositions between CamKIIα-mCherry^+^ CeA projections and GlyT2^+^ PnC neurons (Fig. 7a-c; *N* = 6). In addition, PSD-95, a post-synaptic protein at excitatory synapses, co-localized with these appositions confirming that CamKIIα-mCherry^+^ CeA fibers form excitatory synapses with GlyT2^+^ PnC neurons (Fig. 7c).

**Fig. 8:**
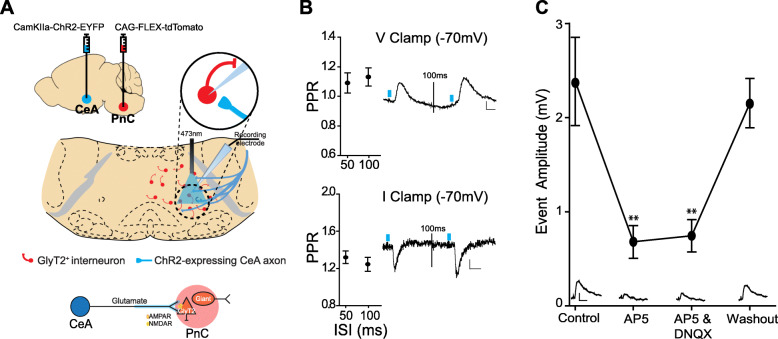
CeA glutamatergic inputs activate GlyT2^+^ PnC neurons via AMPA and NMDA receptors. **a** *Top*, Injection of AAVDJ-CamKIIα-ChR2-eYFP in the CeA and injection of Cre-dependent AAVDJ-tdTomato in the PnC of GlyT2-Cre mice, followed by in vitro patch clamp recordings. *Bottom*, Schematic of the hypothesis being tested. **b** Paired-pulse ratios of the light-evoked EPSPs (*top*) and EPSCs (*bottom*) at 50- and 100-ms interstimulus intervals (ISIs). *Insets*: Representative traces. **c** Graph showing the amplitude of the light-evoked EPSPs recorded in GlyT2^+^ PnC neurons in control, during the sequential bath application of AP5 and DNQX and following washout. *Insets*: Sample traces. Scale: 10 mV/15 ms. Representative of *N* = 10 mice, *n* = 38 neurons. Data are represented as mean ± SEM. *P > 0.05, **P > 0.01. Scale bars: **b** Voltage traces: 2 mV/10 ms; Current traces: 5 pA/5 ms

Next, we recorded basic electrical properties of CamKIIα-eYFP^+^ CeA neurons expressing ChR2 while confirming their sensitivity to blue light photo-stimulation (*N* = 10 mice; *n* = 26 neurons). For this, in vitro patch clamp recordings were performed in acute CeA slices of mice expressing the Cre recombinase enzyme in GlyT2^+^ neurons (i.e., GlyT2-Cre mice). These GlyT2-Cre mice were previously injected with the AAVDJ-CamKIIα-ChR2-eYFP viral vector in the CeA and a Cre-dependent tdTomato viral vector in the PnC, to transduce CeA excitatory cells with ChR2 and GlyT2^+^ PnC neurons with tdTomato, respectively (Fig. 8a and Additional file 1: Figure S5A). Spontaneous excitatory post-synaptic currents (sEPSC) with a mean amplitude of 13.26 ± 0.1 pA were recorded in CamKIIα-eYFP^+^ CeA neurons held at − 70 mV, and current injections elicited action potentials firing at a maximum rate of 14.6 ± 5.4 Hz (Additional file 1: Figure S5C). More importantly, photo-stimulation induced large current responses (821.15 ± 20.3 pA maximum amplitude), indicating that our stimulation protocol successfully activated CamKIIα-eYFP^+^ CeA neurons expressing ChR2 (Additional file 1: Figure S5D).

We then used acute PnC slices obtained from the same mice to determine whether photo-stimulation of CeA excitatory fibers could elicit excitatory synaptic responses in GlyT2^+^ PnC neurons (Fig. 8a). Photo-stimulation (i.e., blue light pulses) evoked excitatory post-synaptic potentials (EPSPs) and currents (EPSCs) in tdTomato-expressing GlyT2^+^ neurons held at − 70 mV. These excitatory responses showed facilitation at ISI of 50 and 100 ms (Fig. 8b, EPSP PPR: 50 ms = 1.09, 100 ms = 1.13; EPSC PPR: 50 ms = 1.318, 100 ms = 1.243). Then, we applied glutamate receptor antagonists to functionally identify the post-synaptic receptors underlying the EPSPs (2.39 ± 0.47 mV) elicited in GlyT2^+^ PnC neurons, in response to the CeA fibers photo-stimulation. AP5 (50 μM) eliminated the NMDAR-dependent component of the EPSP (AP5: 0.68 ± 0.17 mV; 1-way RM ANOVA, F = 9.463, *p* < 0.01), and DNQX (20 μM) blocked the AMPAR-dependent component (Fig. 8c; DNQX: 0.75 ± 0.17 mV; 1-way RM ANOVA, F = 6.009, *p* < 0.01) which recovered by washing out the drugs (2.15 ± 0.75 mV; Fig. 8c).

**Fig. 9:**
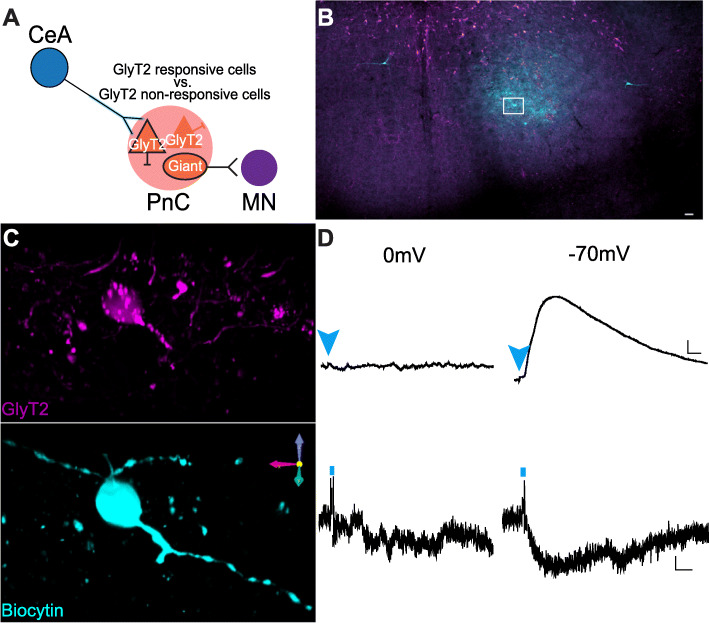
Electrophysiological properties of GlyT2^+^ PnC neurons. **a** Schematic of the hypothesis being tested. **b** Representative PnC slice showing eGFP^+^ fluorescence (magenta) and Biocytin staining (cyan). **c** Higher magnification of the box area in **a**, showing representative morphological reconstructions of recorded GlyT2^+^ cell bodies filled with biocytin. **d** Representative light-evoked voltage (top two traces) and current traces (bottom two traces) recorded at 0 mV (left) and − 70 mV (right) of a GlyT2^+^ neuron responsive to blue light. Blue arrowheads and short vertical lines indicate blue light photo-stimulation. Representative of *N* = 10 mice, *n* = 38 neurons. Scale bars: **b** 250 μm. **c** 10 μm. **d** Voltage traces: 1 mV/10 ms; Current traces: 5 pA/1 ms

Next, to determine if the electrical properties of GlyT2^+^ PnC neurons targeted by CeA excitatory inputs differ from neighboring untargeted GlyT2^+^ PnC cells (Fig. 9a), we compared the intrinsic and spontaneous synaptic properties of tdTomato-expressing GlyT2^+^ neurons (Additional file 1: Figure S6 and Table S1) responsive to light vs. tdTomato-expressing GlyT2^+^ neurons unresponsive to light, at − 70 mV (*N* = 10 mice). Their anatomical location was confirmed post-recording using GlyT2 and Biocytin co-immunostaining, followed by a 3D reconstruction (Fig. 9b,c). As expected from our tract-tracing results, GlyT2^+^ cells medial to the 7th cranial nerve (*n* = 6) and cells located lateroventrally were responsive to blue light (*n* = 12; Fig. 9b,c) whereas cells located along the midline (*n* = 16) or contralateral to the injection site (*n* = 4) did not respond to light. The EPSPs and EPSCs recorded at − 70 mV in light-responsive cells were abolished at 0 mV (i.e., the EPSP reversal potential). None of the light-responsive cells showed IPSP or IPSCs at 0 mV, confirming that no inhibitory inputs were activated by blue light (*n* = 12; Fig. 9d). While both cell types displayed similar passive and active membrane properties (Additional file 1: Figure S6 and Table S1) (F = 1.119, *p* = 0.327; Additional file 1: Figure S6C) and received spontaneous excitatory and inhibitory inputs, the amplitude of sEPSCs (*n* = 18, t = 2.538, *p* = 0.011) and sIPSCs (t = 2.434, *p* = 0.025) of GlyT2^+^ responsive cells was greater compared to that of GlyT2^+^ unresponsive cells (*n* = 20; Additional file 1: Figure S6-B).

Results of these electrophysiological experiments confirm our anatomical data showing that CeA glutamatergic projections activate a subset of GlyT2^+^ PnC neurons located lateroventrally, via AMPA and NMDA receptors. Spontaneous IPSCs were recorded in GlyT2^+^ PnC neurons held at 0 mV (EPSP reversal potential), confirming that these neurons receive inhibitory inputs. However, since blue light photo-stimulation failed to evoke IPSPs in GlyT2^+^ PnC neurons, these results confirm that CamKIIα^+^ CeA neurons do not send inhibitory projections to GlyT2^+^ PnC neurons.

### Optogenetic inhibition of GlyT2^+^ PnC neurons during acoustic prepulses decreases PPI

**Fig. 10:**
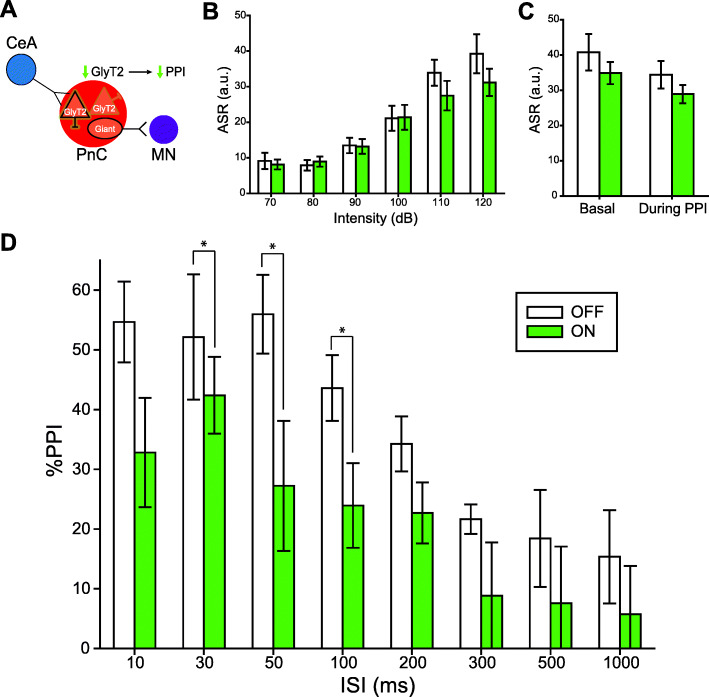
Silencing GlyT2^+^ PnC neurons during acoustic prepulses decreases PPI. **a** Schematic of the hypothesis being tested in GlyT2-Cre mice injected with a Cre-dependent AAV encoding Archaerhodopsin-eYFP (Arch3.0-eYFP). **b** Graph showing no significant effect of green light paired with 70–120 dB acoustic startling pulses on basal startle amplitude [light: (F_(1,7)_ = 1.407, *p* = 0.274); intensity × light interaction:(F_(3,21)_ = 1.747, *p* = 0.188)]. **c** Graph showing no significant main effect of light during 120 dB pulses presented before (basal) vs. randomly during the PPI task, on mean baseline startle amplitude (F_(1)_ = 3.124, *p* = 0.105). **d** Graph showing that green light paired with acoustic prepulses significantly decreased PPI in mice injected with Arch3.0, at ISIs between 30 and 100 ms. We found a significant effect of ISI (F_(6,42)_ = 8.957, *p* < 0.001) and light (F_(1,7)_ = 8.216, *p* = 0.024) (two-way RM ANOVA). *N* = 8 mice per group. Data are represented as mean ± SEM. *p < 0.05, **p < 0.01

Finally, to test whether GlyT2^+^ PnC neurons (likely activated by CeA inputs) contribute to PPI (Fig. 10a), we studied the behavioral contribution of GlyT2^+^ PnC neurons, using GlyT2-Cre mice (*N* = 8 mice) injected with the Cre-dependent optogenetic viral vector rAAVDJ/Ef1α-DIO-eArch3.0-eYFP to transduce GlyT2^+^ PnC neurons with Archaerhodopsin-3 (Arch3.0). We optogenetically inhibited these neurons during PPI through unilateral optic fibers, chronically implanted in the PnC. Photo-inhibition of GlyT2^+^ PnC neurons using 1-ms pulses of green light stimulation presented at 5 Hz had no impact on the acoustic startle response (Fig. 10b) or acoustic pulse-alone stimulations interspersed with PPI trials (Fig. 10c). However, photo-inhibition of GlyT2^+^ PnC neurons during the prepulses and the interpulse intervals significantly decreased PPI by 37–40% at ISIs between 30–100 ms (Fig. 10d).

## Discussion

Overall, our results confirm that in mice, the PnC receives CeA glutamatergic projections which course predominantly ipsilaterally, within the ventrolateral portion of the PnC. Since photo-activating these excitatory projections at the level of the PnC induced a partial PPI and photo-silencing this connection produced an ISI-dependent reduction in PPI, this suggests that CeA glutamatergic inputs contribute to PPI. We also found that the GlyT2^+^ PnC neurons responsive to the photo-stimulation of CeA glutamatergic inputs display AMPA and NMDA receptor-dependent excitatory responses. Finally, our results show that silencing GlyT2^+^ PnC neurons decreases PPI. Together, the influence of CeA glutamatergic neurons in PPI and their direct input to GlyT2^+^ neurons, located at the core of the PnC startle circuit, strongly argue that CeA glutamatergic neurons and GlyT2^+^ neurons are an intrinsic part of the neuronal mechanism regulating the prepulse inhibition of the startle response.

### Anatomical studies

Our data confirm that in mice, there is a direct pathway originating from the CeA onto the pontine reticular formation, as previously described in rats, cats, guinea pigs, and monkeys [55, 58–62]. Our retrograde tracing experiments revealed that afferent projections to the PnC originate in various brain regions including the PPTg and the CeA (Fig. 2 and Additional file 1: Figure S1). Recent evidence in rats and fish suggest that glutamatergic (and likely also, GABAergic cells), but *not* cholinergic neurons, in the PPTg are essential for inhibiting the startle response, during PPI [26–30]. Here, we focused on the glutamatergic projections from the CeA because this region was found to be both relevant for the modulation of the acoustic startle response [43–45] and relevant to diseases associated with sensorimotor gating deficits [48]. In rats, while PnC-projecting CeA neurons were shown to be able to enhance startle reactivity of PnC giant neurons, immunohistochemical assays revealed their non-GABAergic nature [63]. In contrast to the well described CeA inhibitory neuronal populations [54], very few immunohistochemical studies have investigated the population of CeA neurons expressing the glutamatergic neuronal marker VGLUT2^+^, which directly project to pontine neurons. Fung et al. (2011) reported that 24% of all retrogradely labeled CeA neurons that project directly to the oral pontine reticular nuclei (PnO; rostral to the PnC) are VGLUT2^+^ and are located in the lateral and capsular subdivisions of the CeA. Although the studies of Fung et al. (2011) were conducted in guinea pigs, our anatomical data obtained in mice are similar: that is, our CamKIIα-driven tract-tracing analysis and in situ hybridization results (Figs. 3 and 4) also confirm that 80% of the CamKII^+^ CeA neurons virally targeted are glutamatergic and project to the PnC. Interestingly, we demonstrate that amygdalar cell bodies sending projections to the PnC are confined to the medial CeA, and no cell bodies in the lateral and capsular CeA were detected.

Our anterograde tracing experiments and histological analyses also show that the descending CeA glutamatergic fibers course into the ventrolateral part of the PnC, adjacent to the 7th nerve fibers and the olivary complex, in mice (Fig. 3f). Our findings are in accordance with the results of previous anatomical studies in rats and cats showing that descending CeA projecting neurons innervate, directly, with ipsilateral predominance, neurons in the PnC [58–62]. The lateroventral PnC is innervated by cochlear nuclei fibers conveying acoustic startle input to PnC neurons, as lesions in the lateroventral PnC greatly attenuate the startle reflex [19, 20, 64]. Altogether these findings confirm that the CeA projects to a region in the PnC essential for acoustic startle processing.

Interestingly, previous analysis had shown that CeA terminal fibers were only occasionally seen close to the *dendrites* of PnC giant neurons responsible for the startle response [36], in contrast to projections from the cochlear nucleus, which terminate on the *somata* and proximal dendrites of these PnC giant neurons [65, 66]. Rather, such CeA fibers mainly terminated close to the *somata* of small- and medium-sized neurons of the PnC, whose chemical phenotype was not identified [43]. The small diameter (10–20 μm) GlyT2^+^ PnC neurons we focused on in the present study seem to fit that description [47, 50].

Morphological analyses performed in mice expressing eGFP in GlyT2^+^ glycinergic neurons revealed that most of these small diameter neurons are in the brainstem, intermingled with giant neurons in the PnC and the PnO [49, 50]. Notably, the projections of these GlyT2^+^ PnC neurons are distributed similarly in the thalamus of mice and man [67]. Previous experiments in rat brain slices showed that PnC glycinergic interneurons are not activated by the stimulation of afferent sensory fibers within the primary startle pathway [51]. Instead, it was suggested that glycinergic interneurons and glycinergic fibers present within the PnC are most likely under the control of excitatory and inhibitory projections from the midbrain and higher brain structures that modulate the startle responses. Here, our morphological reconstruction data (Fig. 7) provide evidence that the amygdala is one of the brain regions that can activate GlyT2^+^ PnC neurons. Since PnC giant neurons in rodents [51, 68] and humans [69] strongly express glycine receptors, we speculate that once activated by the CeA, GlyT2^+^ neurons reduce the excitability of downstream neurons important for PPI including (but not restricted to) giant PnC neurons.

### Behavioral studies

The acoustic startle response can be modulated in various ways. In our current study, the acoustic startle response is *decreased* by a non-startling stimulus preceding the startle stimulus, resulting in a PPI effect. To better understand the different mechanisms involved in modulating the acoustic startle response, it is important to characterize the inputs to PnC neurons at the core of the primary startle pathway and their sensorimotor effects. Recently, functional imaging studies and c-Fos expression data in rats provided strong evidence that CeA neuronal activity is increased during PPI [48]. The objective of our behavioral experiments was to demonstrate the contribution of an CeA-PnC excitatory pathway in reducing the acoustic startle reflex, and its relevance to PPI. We hypothesized that if CeA inputs to the PnC modulate startle during PPI, they would need to be activated prior to a startle response. We validated our hypothesis in vivo and we showed that unilateral [20, 70–73] photo-inhibition of the CeA-PnC glutamatergic connection during the interval between the prepulse and the pulse decreases PPI (Fig. 5). Interestingly, photo-inhibition of the CeA-PnC glutamatergic connection did not modify an ongoing startle response elicited by a pulse-alone startling stimulation (i.e., in the absence of a prepulse). Thus, our data suggest that in the context of PPI, silencing CeA glutamatergic neurons prior to a startling stimulation reduces PPI but does not affect baseline startle elicited by a pulse-alone stimulation.

During conditioned and unconditioned states of fear, CeA stimulation can *amplify* the acoustic startle response [44–46]. Interestingly, our results showing the role of the CeA during PPI are consistent with the modulatory role of the CeA in fear studies demonstrating that lesions of the CeA block fear *potentiation* of startle without blocking the acoustic startle response itself [57].

In an attempt to mimic an acoustic prepulse and further demonstrate the behavioral relevance of this pathway, we photo-activated CeA-PnC glutamatergic connection prior to an acoustic startling stimulation (Fig. 6). This led to a lower “PPI-like” effect than using an acoustic prepulse. The fact that photo-activation of CeA glutamatergic fibers partially mirrored the effects of acoustic prepulses suggest that CeA excitatory inputs to the PnC do not regulate PPI alone, but work alongside other neuronal elements and pathways, including PPTg-dependent mechanisms. It should also be mentioned that, under physiological conditions, CeA neuronal firing might not necessarily follow the stimulation frequency we used. Despite this, our results clearly show that CeA-PnC excitatory inputs contribute to PPI. Another concern is the possibility that optogenetic inhibition at presynaptic terminals produces unwanted technical effects including a paradoxical increase in neurotransmitter release. To avoid this possibility, we used precisely timed, repetitive light stimulation instead of sustained archaerhodopsin and halorhodopsin activation, which previously led to an increase in spontaneous release [74], allowing us to investigate in vivo physiological conditions more closely.

Aside from their role in fear-potentiated startle, no study has directly investigated the function of CeA glutamatergic neurons projecting to the PnC, including GlyT2^+^ neurons. At the level of the PnO, CeA glutamatergic descending projections were suggested to contribute to rapid eye movement (REM)/active sleep by activating large (i.e., cell body of ~ 30 μm), presumably glutamatergic “REM-on” giant neurons [75]. Since CeA glutamatergic projections target both giant neurons [43] and GlyT2^+^ neurons (present study) in the PnC as well as giant neurons in the PnO [75], it is likely that these projections also target GlyT2^+^ neurons in the PnO. Interestingly, the activation of these two reticular cell types (i.e., giant neurons and GlyT2^+^ cells) leads to opposite behavioral outcomes. That is, in the PnO, giant neurons contribute to REM sleep and GlyT2^+^ neurons are crucial for awake cortical activity [67]. In the PnC, giant neurons are responsible for startle responses [47], and GlyT2^+^ neurons are crucial for behavioral arrest [67]. Our data show that silencing GlyT2^+^ neurons significantly reduced PPI at short interstimulus intervals between the prepulse and the pulse. Based on our data and that of other groups, GlyT2^+^ PnC neurons activated by CeA glutamatergic neurons are likely the ones that inhibit startle during PPI. Since our results suggest that CeA-PnC excitatory synapses can regulate PPI at ISI between 30 and 300 ms, it is tempting to speculate that during PPI elicited at these ISIs, CeA glutamate neurotransmission has sufficient time to activate GlyT2+ PnC neurons, leading to a feedforward inhibition. Future experiments should be done to identify the post-synaptic targets of GlyT2^+^ PnC neurons that are involved in PPI. The amygdala, including the CeA, comprises a wide array of molecularly, electrophysiologically, and functionally distinct cell populations [54] that were shown to play differential roles in fear and extinction learning. Therefore, it is possible that a subset of CeA glutamatergic neurons activates a group of lateroventral GlyT2^+^ PnC neurons, leading to PPI, whereas another subset of CeA glutamatergic neurons activates giant PnC neurons, leading to enhanced fear [76] (see schematic of Fig. 7a).

### Electrophysiological studies

Previous studies aiming to describe the electrophysiological effect of activating CeA glutamatergic neurons used electrical stimulating electrodes, making it impossible to distinguish whether fibers of passage or CeA cell bodies were activated. Moreover, pharmacological activation of CeA neurons did not allow to selectively activate excitatory neurons [43]. To reconcile the glutamatergic nature of the CeA neurons projecting to the PnC with their contribution to an inhibitory phenomenon, here, we performed in vitro patch clamp recordings in GlyT2-Cre mice (Fig. 8). We recorded EPSPs in GlyT2^+^ PnC neurons labeled with tdTomato, in response to targeted activation of CeA glutamatergic inputs expressing ChR2. Altogether, our results demonstrate that 1-CeA glutamatergic projections activate GlyT2^+^ PnC neurons via AMPA and NMDA receptors, and 2-CeA-PnC glutamatergic synapses display short-term synaptic facilitation.

Previous rat pharmacological in vivo studies showed that blocking AMPA and NMDA receptors in the PnC inhibits acoustic startle responses [77–79]. Interestingly, our behavioral data suggest that blocking a glutamatergic connection between the CeA and the PnC reduces PPI. Our PPI in vivo results do not conflict with these previous studies showing the important role of the glutamate neurotransmission within the PnC startle pathway. Our in vivo results clearly indicate that during PPI, inhibition of startle involves CeA glutamatergic cells directly projecting to the PnC. In that context, acoustic prepulses activate glutamatergic receptors located on GlyT2^+^ and, likely also, PnC giant neurons. One hypothesis is that during PPI, CeA glutamate neurotransmission activates GlyT2^+^ PnC neurons leading to a feedforward inhibition. This hypothesis is clearly supported by our in vitro electrophysiological recordings in PnC slices showing that AMPA/NMDA receptor blockers reduce the EPSPs in GlyT2^+^ PnC neurons elicited by CeA glutamatergic fiber stimulation (Fig. 8c).

Paired photo-stimulation at CeA-PnC glutamatergic synapses elicited short-term synaptic facilitation of EPSPs at ISI of 50 and 100 ms (Fig. 8b). Synaptic facilitation reflects presynaptic enhancement of neurotransmitter release, associated with residual calcium accumulation within presynaptic terminals following the first stimulation [80]. Interestingly, the synaptic facilitation we recorded at CeA-PnC glutamatergic synapses and the CeA-dependent PPI values we measured in vivo both occur within similar time scales.

Typically considered as a GABAergic (and non-cholinergic) nucleus, the CeA includes an ensemble of several other neurochemical and neuropeptide profiles, such as galanin, somatostatin, substance P, and corticotropin-releasing factor [81–85]. In fact, the CeA sends GABAergic inputs to several brainstem regions adjacent to the PnC, such as the ventrolateral periaqueductal grey [86], locus coeruleus [87], and nucleus of the solitary tract [88]. Furthermore, altered GABAergic neurotransmission is seen in schizophrenia [89, 90] and is associated with abnormal acoustic startle reflex and PPI [91–95]. GABAergic neurotransmission was previously shown to modulate the startle pathway [96]. Although our data rule out the possibility that the CamKIIα^+^ CeA neurons we targeted are inhibitory (Figs. 4 and 8), future work should determine whether the CeA also contribute to PPI through GABAergic projections. Previous studies have highlighted the importance of pontine glutamatergic and GABAergic signaling during PPI. In addition, PPI is sensitive to changes in glutamatergic and GABAergic transmission in several brain regions, such as the amygdala [37, 38, 91], hippocampus [92], superior colliculus [93], PPTg [29, 30, 94], and nucleus accumbens [95]. These brain regions also exhibit anatomical and functional abnormalities in neuropsychiatric disorders associated with sensorimotor gating deficits. Here, we provide evidence for a potential amygdala-dependent glutamatergic mechanism at the PnC level that could be impaired in diseases associated with PPI deficits.

## Conclusions

Overall, the results presented here along with the body of literature on the role of the amygdala on acoustic startle modulation suggest that CeA excitatory neurons send inputs to the PnC, where they activate a subpopulation of GlyT2^+^ neurons that contribute to PPI. We propose that the primary startle pathway (Fig. 1; red) is modulated by two parallel circuits: (1) the well-established the CSPP network (Fig. 1; light blue and dark blue brain regions/pathways), and (2) the CeA-PnC glutamatergic connection (Fig. 1; dark blue dotted connection delineated by the red dotted square). These two circuits ultimately converge at the level of the PnC. Our results are aligned with recent data obtained by other groups revisiting the theoretical constructs of PPI, describing glutamatergic, GABAergic, and glycinergic underlying mechanisms. More importantly, our results shed light into the basic processes underlying sensorimotor gating, and our disease-relevant proposed circuit should expand insights derived from disease experimental systems.

## Methods

### Mice

Experiments were performed on C57BL/6 male mice (*N* = 61; The Jackson Laboratory, Bar Harbor, ME), GlyT2-eGFP mice (*N* = 6; graciously provided by Dr. Manuel Miranda-Arango, University of Texas at El Paso, El Paso, TX), and GlyT2-Cre^+/−^ mice (*N* = 18; graciously provided by Dr. Jack Feldman, University of California, Los Angeles). Litters were weaned at PND 21 and housed together until stereotaxic microinjections were performed at PND 70–84 (adult). Mice received food and water ad libitum in a 12-h light/dark cycle from 7:00 am to 7:00 pm. This age corresponds to the age of the animals used in the Paxinos and Franklin Mouse Brain Atlas, from which all the stereotaxic coordinates were derived, and cytoarchitectural boundaries delineated [52]. Following surgical procedures, mice were single-housed and monitored for the duration of the recovery period. Experiments were performed in accordance with and approved by the Institutional Animal Care and Use Committee of the University of Texas at El Paso (UTEP) and the University of Massachusetts Amherst (UMass).

### Stereotaxic microinjections

Mice were sedated by inhaling 5% isoflurane vapors (Piramal Critical Care, Bethlehem, PA), then placed on a stereotaxic apparatus (model 900, David Kopf, Tujunga, CA) and immobilized using ear bars and a nose cone. Mice were maintained under 1.5–2% isoflurane throughout the duration of the surgical procedure. With bregma as a reference, the head of the mice were leveled on all 3 axes. A craniotomy was performed directly dorsal to the injection site. Then, using a microinjector (Stoelting Co., Wood Lane, IL) with a 5-μl Hamilton syringe (Hamilton Company Inc., Reno, NV) and a 32-gauge steel needle, unilateral injections of 50–80 nl of the retrograde neuronal tracer Fluoro-Gold (Molecular Probes, Eugene, OR, catalog# H22845, lot# 1611168) were infused into the PnC (coordinates from bregma: AP − 5.35 mm; ML + 0.5 mm, DV − 5.6 mm; *N* = 4 mice). The CAG-FLEX-tdTomato (Addgene# 28306-AAV1; lot# v16602) or rAAVDJ/Ef1α-DIO-eArch3.0-eYFP (Deisseroth Lab, virus# GVVC-AAV-055) viral vectors were injected (200 nl) in the PnC of mice expressing the CRE recombinase enzyme in GlyT2^+^ neurons (GlyT2-Cre mice; *N* = 10). In separate animal cohorts, 100–125 nl of AAV particles were unilaterally injected in the CeA (AP − 1.35 mm, ML + 2.66 mm, DV − 4.6 mm). For these viral injections, pAAV DJ-CamKIIα-eArch3.0-eYFP (Deisseroth Lab, # GVVC-AAV-053, lot# 1668 and 3605), pAAV DJ-CamKIIα-NpHR3.0-eYFP (Deisseroth Lab, #GVVC-AAV-057, lot#1378), pAAV DJ-CamKIIα-hChR2(H134R)-eYFP (Deisseroth Lab, #GVVC-AAV-037, lot#3150), pAAV DJ-CamKIIα-eYFP (Deisseroth Lab, #GVVC-AAV-8), or pAAV DJ-CamKIIα-mCherry (Deisseroth Lab, #GVVC-AAV-009) viral particles were used (4 × 10^12^ particles/mL; vectors were obtained from Dr. Karl Deisseroth’s Lab/Optogenetics Innovation Lab, Gene Vector and Virus Core, Stanford University, Palo Alto, CA, *or* through Addgene from Dr. Edward Boyden’s Lab plasmids from, Massachusetts Institute of Technology, Cambridge, Massachusetts). Fluoro-Gold and viral particles were delivered at a rate of 50 nL/min. The microinjection syringe was left in place for 10 min after infusion to limit spillover during needle retraction. Mice injected with Fluoro-Gold recovered for 5–7 days, to allow optimal Fluoro-Gold retrograde transport to occur. AAV-injected mice recovered for 3–5 weeks to allow sufficient time for maximal viral transduction.

### Immunohistochemistry

Mice were perfused transcardially with 0.9% saline solution for 10 min followed by 4% paraformaldehyde (PFA) in 0.1 M phosphate buffer saline (PBS; pH 7.4) for 15 min, brains were then extracted and post-fixed overnight in 12% sucrose in PFA solution. After three 0.1 M PBS rinses (5 min each), brains were frozen in chilled hexanes for 1 min and stored at − 80 °C. Using a microtome, four 1-in-5 series of 30-μm coronal sections were cut and stored in cryoprotectant (50% 0.05 M phosphate buffer, 30% ethylene glycol, 20% glycerol) at − 20 °C. One of the series was rinsed three times (5mins each) with 0.1 M Tris-buffered saline (TBS; pH 7.4), mounted and coverslipped to visualize injection and projection sites. An adjacent series of brain sections was Nissl-stained to determine plane of section and delineate cytoarchitectural boundaries. The two remaining series were used for immunohistochemistry. For mice injected with Fluoro-Gold, coronal tissue sections at the level of the PnC, CeA, and PPTg were washed with 0.1 M TBS (5 washes, 5 min each) and incubated in blocking solution (2% normal donkey serum, 0.1% Triton X-100; in 0.1 M TBS) for 1–2 h at room temperature. PPTg sections were incubated with a goat anti-ChAT primary antibody (1:100, Millipore, catalog# AB144P-200UL, lot# 2854034, RRID:AB_90661) for 60 h at 4 °C, washed with TBS, and then incubated in a Cy3-conjugated donkey anti-goat secondary antibody (1:500, Jackson ImmunoResearch Laboratories, catalog# 705-165-147, lot# 115611, RRID:AB_2307351) for 4-5 h at room temperature. Tissue slices were then washed with TBS, mounted, and coverslipped. Similarly, for mice injected with viral particles, tissue sections containing the PnC, CeA, and the PPTg were incubated in a chicken anti-GFP primary antibody (1:1000, Abcam, catalog# ab13970, lot# GR236651-13, RRID:AB_300798), then incubated in an Alexa Fluor 488-conjugated donkey anti-chicken secondary antibody (1:500, Jackson ImmunoResearch Laboratories, catalog# 703-545-155, lot# 130357, RRID:AB_2340375), followed by incubation in NeuroTrace^TM^ (640/660 deep red fluorescent nissl stain, 1:100 in TBS, Thermo Fisher, catalog# N21483, RRID:AB_2572212). NeuroTrace^TM^ was alternatively used to determine plane of section and cytoarchitecture. Tissue sections at the level of the PnC of GlyT2-eGFP mice injected with pAAV DJ-CamKIIα-mCherry in the CeA (*N* = 6 mice) were incubated with a chicken anti-mCherry (1:1000, Abcam, catalog# ab205402, lot# GR225123-3, RRID:AB_2722769) and a rabbit anti-PSD95 (1:500, Abcam, catalog# ab12093, lot# GR317630-1, RRID:AB_298846) primary antibodies. Then, sections were incubated with a Cy3-conjugated donkey anti-chicken (1:500, Jackson ImmunoResearch Laboratories, catalog# 703-165-155, lot# 130328, RRID:AB_2340363) and a Cy5-conjugated donkey anti-rabbit (1:500, Jackson ImmunoResearch Laboratories, catalog# 705-545-147, lot#125100, RRID:AB_2336933) as described above.

### In situ hybridization

Mice injected with pAAVDJ-CamKIIα-eYFP in the CeA (*N* = 3 mice) were anesthetized with inhaled isoflurane and rapidly decapitated. Brains were harvested, frozen in chilled isopentane, and stored at – 80 °C. Serial coronal sections (15 μm) at the level of the CeA were cut in a cryostat, directly mounted onto glass slides, and stored at – 80 °C. Tissue sections on slides were submerged in freshly prepared cold 4% PFA for 15 min, rinsed twice briefly with 0.1 M phosphate buffer (PB) and dehydrated in increasing ethanol solutions (50%, 70%, 100%, 100%; 5 min each at room temperature). Then, the RNAscope assay (Advanced Cell Diagnostics) started by incubating in hydrogen peroxide (H_2_O_2_) for 10 min in a humidified box, followed by protease III incubation for 15 min. RNA hybridization probes against genes encoding mouse VGLUT2 (319171-C1) and eYFP (312131-C2) were then incubated for 2 h at 40 °C. Antisense probes were also included as controls in a separate glass slide. Probe signals were then developed separately with Opal Dyes (opal 690 1:1.5 K, opal 520 1:750) and coverslipped with ProLong Gold^TM^ with DAPI.

### Morphological reconstruction

Z-stacks from tissue sections of GlyT2-eGFP and GlyT2-Cre mice were obtained on a Nikon A1 Resonant Confocal microscope (Nikon Instruments Inc., Melville, NY) equipped with NIS-Elements High Content Analysis software (version 5.02, Nikon Instruments Inc., Melville, NY). Tissue sections containing labeled CeA and PnC neurons were first examined on a single Z-plane with the × 10 objective to survey the tissue section. Using a × 60 objective, an area (212.56 μm width × 212.56 μm height) within CeA and PnC sections was then sequentially scanned by the 488-, 561-, and 640-nm laser lines in 0.1 μm Z-steps throughout the 30-μm tissue section. Z-stacks were analyzed with NIS-Elements 5.0 Advanced Research software (version 5.02, Nikon Instruments Inc., Melville, NY). To visualize close appositions of CeA axons (labeled with mCherry) with GlyT2^+^ neurons (labeled with eGFP) in GlyT2-eGFP mice, a binary layer was configured to segregate putative synaptic contacts of > 50 nm in distance (due to technical limitations). These contacts were imaged in split-channels and orthogonal views. Then, z-stacks were reconstructed in three-dimension and volume was rendered.

### Nissl stain

Series of tissue slices were mounted on gelatin-coated slides and air-dried overnight. Slides were immersed in deionized water, followed by ascending concentrations of ethanol (3 min each: 50%, 75%, 95%, and 100%), and then in xylenes (30 min). Brain slices were rehydrated in descending concentrations of ethanol and DI water, dipped 12–20 times in a thionin acetate solution, and then washed in DI water. Brain slices were dehydrated, and slides were then coverslipped with DPX and air-dried overnight.

### Microscopy analysis

Tissue sections were analyzed with an Axio Observer.Z1 epifluorescence microscope (Carl Zeiss Inc., Thornwood, NY) equipped with Fluoro-Gold, GFP, Cy3 filters, × 10 and × 40 objectives, a motorized stage, and Axiovision Rel. 4.8 software (Carl Zeiss Inc., Thornwood, NY). To create photomontages, single Z-plane images were obtained with the MosaiX module of the Axiovision Rel. 4.8 software at × 10 for each fluorophore sequentially (1024 × 1024 pixel resolution). Images acquired for the intensity and quantification of eYFP fluorescence analysis were captured and processed using identical settings. A total of 836 images (fluorescence and bright-field) were analyzed for each brain region. Nissl-stained slices were imaged using bright-field microscopy, and boundaries were delineated using Adobe Illustrator (Adobe, San Jose, CA).

### Electrophysiological recordings

Whole-cell recordings (*N* = 10 mice; *n* = 26 CeA neurons and *n* = 38 GlyT2^+^ neurons) were performed using glass pipettes (3–5 MΩ) filled with intracellular solution (in mM): KMeSO4 (125), KCl (10), HEPES (10), NaCl (4), EGTA (0.1), MgATP (4), Na2GTP (0.3), Phosphocreatine (10), Biocytin (0.1%) (pH = 7.3; osmolarity = 285–300 mosm). The glass microelectrode was mounted on a patch clamp headstage (Molecular Devices LLC, Sunnyvale, CA; catalog# CV-7B), which was attached to a multi-micromanipulator (Sutter Instrument, Novato, CA; catalog# MPC-200). Data were acquired with pClamp10 software using a MultiClamp™ 700B amplifier (Molecular Devices LLC, Sunnyvale, CA) and a Digidata 1550B digitizer (Molecular Devices LLC, Sunnyvale, CA). EYFP-expressing CeA cells and tdTomato-expressing GlyT2^+^PnC cells were imaged and targeted using NIS-Elements Basic Research software (version 5.11, Nikon Instruments Inc., Melville, NY). Only cells with an initial seal resistance greater than 1GΩ, a resting membrane potential between − 60 mV and − 70 mV, and a holding current within – 100 pA to 100 pA at resting membrane potential and overshooting action potentials were used.

In CeA slices, 15 pA depolarizing current steps were injected for 500 ms to induce action potentials in CeA neurons expressing CamKIIα-ChR2-eYFP, in the current clamp. Spontaneous EPSCs were recorded at a holding potential of − 70 mV, in the voltage clamp. Evoked EPSPs were also recorded in these CeA neurons held at − 70 mV, in response to a 1-ms blue light pulse. Blue light was delivered every 30 s using a 200-μm optic fiber mounted on a micromanipulator connected to a blue LED (473 nm; Plexon, Dallas, TX) and positioned in close proximity to the recorded neuron.

In PnC slices, electrical properties of the GlyT2^+^ neurons were first recorded in the voltage clamp. Spontaneous excitatory post-synaptic currents (sEPSC) were recorded for 5 min at − 70 mV, and inhibitory post-synaptic currents (IPSCs) were recorded for 5 min at 0 mV. Then, in the current clamp mode, 15 pA depolarizing current steps (from – 150 pA to 150 pA) were injected for 500 ms to analyze the spiking properties of GlyT2^+^ cells. Pulses of blue light (1 ms), applied every 30 s, were used to photo-stimulate CeA excitatory fibers in PnC slices. The photo-stimulation elicited EPSPs and EPSCs in GlyT2^+^ neurons, held at − 70 mV. Paired light pulses with 50 and 100 ms ISI were also delivered to characterize short-term plasticity. GlyT2^+^ neurons were then held at 0 mV, to record light-evoked inhibitory post-synaptic current (IPSCs) or potentials (IPSPs). The NMDA receptor antagonist AP5 (50 μM) and the AMPA receptor antagonist DNQX (25 μM) were freshly diluted prior to use. At synapses between CeA excitatory cells and GlyT2^+^ neurons, synaptic events were recorded for 10 min in aCSF. Then, 20 min after the bath application of glutamate receptor antagonists, synaptic events were recorded during 10 min in the presence of the antagonists. This was followed by a 20-min washout period, and synaptic events were recorded during the following 10 min, in aCSF.

At the end of all whole-cell recordings, the cell membrane was sealed by forming an outside-out patch. The glass microelectrode was slowly retracted, and as the series resistance increased, the membrane potential was clamped at − 40 mV. The 300-μm-thick acute brain slices containing the recorded cells (CeA or PnC) were immersed in 4% PFA solution overnight. Following overnight PFA fixation, these brain slices were rinsed with PBS (3 times, 5 min each). Slices were then incubated in anti-RFP and/or anti-GFP antibodies and in complementary secondary antibodies to enhance the fluorescence of the viral vectors used. Following PBS rinses, slices were incubated with Cy5-conjugated streptavidin (a biotin-binding protein) diluted in PBS (with 0.1% Triton X-100) at room temperature for 4–5 h or overnight at 4 °C. Slices were then rinsed with PBS, mounted on glass slides, coverslipped, and sealed with ProLong^TM^ Gold antifade reagent (Invitrogen by Thermo Fisher Scientific, Waltham, MA, catalog# P36934, lot# 1943081), and air-dried overnight in the dark.

### Behavioral testing

Three to four weeks after the viral injection in the CeA, non-injected WT control mice and WT mice injected with a viral vector were sedated by inhaling 5% isoflurane vapors, placed in a stereotaxic apparatus, and immobilized using ear bars and a nose cone. Mice were maintained under anesthesia (1.5–2% isoflurane), and the head was leveled in all three axes. With bregma as a reference, a craniotomy was drilled directly dorsal to the implantation site, at the PnC level. A cannula guide with a 200-μm core optical fiber (Thorlabs, Newton, NJ) was then implanted over the PnC (AP − 5.35 mm, ML + 0.5 mm, DV − 5.3 mm), and cemented to the skull with dental cement (Parkell, Edgewood, NY). Mice recovered for 7 days post-surgery before behavioral testing. Mice underwent the PPI task in a startle response system (PanLab System, Harvard Apparatus, Holliston, MA). Behavioral testing trials were designed, and data were recorded using PACKWIN V2.0 software (Harvard Apparatus, Holliston, MA). Sound pressure levels were calibrated using a standard SPL meter (model 407730, Extech, Nashua, NH). Mice were placed on a movement-sensitive platform. Vertical displacements of the platform induced by startle responses were converted into a voltage trace by a piezoelectric transducer located underneath the platform. Startle amplitude was measured as the peak to peak maximum startle magnitude of the signal measured during a 1-s window following the presentation of the acoustic stimulation. Prior to any testing session, animals were first handled and acclimatized to the testing chamber, where the mice were presented to a 65-dB background noise, for 10 min. This acclimatization period was used to reduce the occurrence of movement and artifacts throughout testing trials. Following the acclimatization period, an input/output (I/O) assay was performed to test startle reactivity. This I/O test began with the presentation of a 40-ms sound at different intensities (in dB: 70, 80, 90, 100, 110, and 120) every 15 s, in a pseudorandomized order. Background noise (65 dB) was presented during the 15 s between sounds. A total of 35 trials (i.e., 7 sound intensities, each sound presented 5 times) were acquired and quantified. Startle reactivity, derived from this I/O assay, allowed the gain of the movement-sensitive platform to be set. This gain allowed the startle responses to be detected within a measurable range. Once determined, the gain for each experimental subject was kept constant throughout the remaining of the experiment. Following a 1-h resting period, mice were presented with seven startle-inducing 120 dB (40 ms) sounds called “pulse-alone” stimulations. These 120 dB sounds were presented every 29 s (interspersed with 65 dB background noise) and were used to achieve a stable baseline startle response. The following PPI test consisted of two different conditions as follows: (1) startling pulse-alone stimulations (for baseline startle amplitude), and (2) combinations of a prepulse (75 dB noise; 20 ms) followed a 120-dB startling pulse (40 ms) at 8 different interstimulus intervals (in ms): 10, 30, 50, 100, 200, 300, 500, and 1000 (end of prepulse to onset of startle pulse). The inter-trial interval of these two conditions was 29 s.

For combined optogenetic manipulations, animals injected with either control viral vectors or vectors containing ChR2 (*N* = 8 mice), Arch3.0 (*N* = 16 mice), NpHR3.0 (*N* = 8 mice), or the control vector (pAAV DJ-CamKIIα-eYFP; *N* = 8 mice) were tested in the startle chamber. These animals were closely monitored to ensure that they were comfortably tethered to an optic fiber, which exited through a small opening from the roof of the startle chamber. The optic fiber (200 μm diameter, Thorlabs, Newton, NJ) was connected to the animal’s head via a cannula implanted on the head of the mouse with a zirconia sleeve (Thorlabs, Newton, NJ). Animals were tethered ~ 15 min before testing and allowed to move freely, exploring their home cages before being transferred to the startle chamber. Optogenetic stimulation was triggered by a signal from the Packwin software (PanLab System; Harvard Apparatus, Holliston, MA), which was transformed into a TTL pulse. This TTL pulse triggered a waveform generator (DG1022, Rigol Technologies), which was used to modulate light stimulation. Photo-stimulation was delivered using a blue 473-nm laser (Opto Engine LLC, Midvale, UT) for ChR2 activation. Photo-inhibition was delivered using a yellow 593.5-nm laser (Opto Engine LLC, Midvale, UT) for NpHR3.0 activation or a green 532-nm LED (Plexon, Dallas, TX) for Arch3.0 activation. During PPI trials paired with optogenetic inhibition, a train of light stimulation (1 ms light ON, 200 ms light OFF) was delivered at 5 Hz and was either (1) delivered 500 ms prior to and concurrent to the pulse-alone stimulation, or (2) delivered 500 ms before the prepulse, lasting the entire ISI. During PPI trials with optical stimulation used as a prepulse, a 5-Hz or 20-Hz stimulation train (3 pulses of 15 ms) was delivered at various ISI (10, 30, 50, 100, 200, 300, 500, and 1000 ms; end of prepulse to onset of startle pulse) prior to the startling pulse. Blue light stimulation was paired with pulse-alone stimulations in a subset of mice. At the end of each experiment, histological analyses were performed to confirm that (1) the injected viral particles were confined to the CeA, and (2) the cannula guide placement was successfully aimed at the PnC. If these criteria were not met, the subject was excluded from the study.

### Statistical analysis

Cell counting of EYFP-labeled or VGLUT2-expressing somata within the CeA was performed in a tissue slice series of 6 slices spanning levels 40 to 44 of the Paxinos and Franklin Mouse Brain Atlas [52]. Imaging was performed as outlined in the “Microscopy analysis” section. Percentages of labeled somata were calculated as EYFP+/Neurotrace-labeled cells (Fig. 3) or VGLUT2+/EYFP+ (Fig. 4). Statistical analyses were performed using SigmaPlot (Systat Software, Inc., San Jose, CA). Normality and equal variance of the data were first tested, and data transformations were made before performing further statistical analyses. We determined the significance of the interaction between the factors assessed using ANOVA. For the results of whole-cell patch clamp recordings with receptor antagonists, one-way repeated-measures (RM) ANOVA and Tukey post hoc testing were used to assess the effect of the receptor antagonists on the light-evoked events. For PPI in vitro results, one-way ANOVAs and Tukey post hoc testing were used to reveal if at any ISI the electrically evoked fEPSPs were significantly attenuated by the optical stimulation of CeA-PnC excitatory synapses. PPI was defined and measured as [1–(startle amplitude during “Prepulse+Pulse” trials/startle amplitude during “Pulse” trials)] × 100. Two-way RM ANOVA was used to assess the effect of the vector used, light, sound intensity/ISI, and light interaction and the interaction among groups. Then Tukey testing was applied for post hoc comparisons. For optical stimulation experiments where the photo-stimulation of CeA fibers was used as a prepulse in vivo, two-way RM ANOVA was used to assess the effect of the stimulation modality/frequency used, ISI, ISI, and stimulation modality/frequency interaction and the interaction among groups. Then, Tukey testing was applied for post hoc comparisons. A confidence level of *p* < 0.05 was considered statistically significant. Sample sizes were chosen based on expected outcomes, variances, and power analysis. Data are presented as means ± SEM. N indicates total number of animals; n indicates total number of brain slices or testing trials. Adobe Illustrator was used to create figures.

## Supplementary Information

**Additional file 1: Figure S1.** The PnC receives monosynaptic inputs from the PPTg. (A) Representative PPTg coronal section showing the immunofluorescence of the cholinergic marker ChAT (magenta), which delineates the PPTg. (B) PPTg ChAT^+^ cell bodies (magenta) shown at higher magnification. (C) Representative PPTg section showing Fluoro-Gold staining (green). (D) Overlay of B and C, representative co-immunostaining of Fluoro-Gold and ChAT fluorescence. Arrows indicate neurons stained with Fluoro-Gold that are non-cholinergic (ChAT^-^). Arrowheads indicate neurons stained with Fluoro-Gold that are ChAT^+^. Representative of *N* = 4 mice. Scale bars: (A) 500 μm, (B-D) 100 μm. **Figure S2.** CamKIIα^+^ CeA projection neurons are non-GABAergic. (A) Representative images of GABA (magenta) immunostaining in the medial division of the CeA. Arrowheads indicate GABA^+^ CeA cell bodies. (B) Representative image of eYFP fluorescence (green). Arrows indicate CeA cell bodies and neurites. (C) Overlay of A and B, representative co-immunostaining for eYFP and GABA showing no overlap. Representative of *N* = 4 mice. Scale bars: (A-C) 250 μm. **Figure S3.** NpHR3.0 inhibition of the CeA-PnC excitatory connection reduces PPI. (A) Graph showing no significant main effect of yellow light on mean baseline startle amplitude [sound: (F_(1,11)_ = 1.935, *p* = 0.115); yellow light: (F_(1)_ = 0.00297, *p* = 0.958); sound intensity*light interaction (F_(1,6)_ = 0.102, *p* = 0.996)] by comparing non-injected WT control mice, mice injected with eYFP only (light ON or OFF) and mice injected with Halorhodopsin (NpHR3.0; light ON or OFF). (B) Graph showing no significant main effect of light during 120 dB pulses presented before (basal) vs. randomly during the PPI task, on mean baseline startle amplitude among animal groups (F_(1)_ = 0.394, *p* = 0.543). (C) Graph showing that optogenetic silencing of CeA-PnC excitatory synapses during prepulses significantly decreased PPI in mice injected with NpHR3.0 at ISIs between 50 and 300 ms. We found a significant effect of ISI (F_(1,7)_ = 33.019, *p* < 0.001), light (F_(1)_ = 5.371, *p* = 0.041) and the light*ISI interaction (F_(1,7)_ = 3.692, *p* = 0.002) on PPI (ANOVA). *N* = 8 mice per group. Data are represented as mean ± SEM. *p < 0.05, **p < 0.01. **Figure S4.** Photo-stimulating CeA-PnC glutamatergic synapses does not alter acoustic PPI. (A) Graph showing no significant main effect of blue light on mean baseline acoustic startle amplitude in mice injected with ChR2 (light ON or OFF). We found no effect of viral vector type (F_(1,2)_ = 1.417, *p* = 0.247) or viral vector*sound intensity interaction (F_(1,12)_ = 0.413, *p* = 0.956). (B) Graph showing no significant main effect of blue light paired with acoustic prepulses on PPI, at all ISIs tested (F_(1,14)_ = 0.151, *p* = 1.000). *N* = 6 mice. Data are represented as mean ± SEM. **Figure S5.** Intrinsic and synaptic properties of CamKIIα-eYFP^+^ CeA glutamatergic neurons expressing ChR2. (A) Scheme of the *in vitro* patch clamp recording experiment showing the injection site of AAVDJ-CamKIIα-ChR2-eYFP and the recording of CeA neurons of GlyT2-Cre mice. (B) Plot showing the cumulative distribution of sEPSC amplitude. *Inset*, representative trace. (C) Plot of the firing rate as a function of depolarizing currents. *Inset*, representative traces. (D) Input/output curve of light-evoked current. (E) Three-dimensional reconstruction representative of a recorded CeA neuron filled with biocytin. *N* = 10 mice, *n* = 26 neurons. Data represented as mean ± SEM. *P > 0.05, **P > 0.01. **Figure S6.** Intrinsic and synaptic properties of GlyT2^+^ PnC neurons. (A) Plot showing the cumulative distribution of sEPSC amplitude recorded in GlyT2^+^ cells unresponsive (*Top*; *n* = 20 cells) and GlyT2^+^ cells responsive (*Middle*; *n* = 18) to light. *Bottom*: cumulative sEPSC distribution plots. (B) Plot showing the cumulative distribution of sIPSC of GlyT2^+^ cells unresponsive (*Top*; *n* = 20 cells) and GlyT2^+^ cells responsive (*Middle*; *n* = 18) to light. *Bottom*: cumulative sIPSC distribution plots. (C) Graph showing no significant difference between the firing rate and threshold current to elicit APs in GlyT2^+^ cells unresponsive and responsive to light. *Insets*: Representative traces of *N* = 10 mice, *n* = 38 neurons. Data represented as mean ± SEM. **Table S1.** Passive membrane properties of PnC GlyT2 cells. Statistical analysis showed no significant differences in the access resistance (R_a_), membrane resistance (R_m_), membrane capacitance (C_m_), time constant (τ) and holding current (I_h_) of light responsive and unresponsive GlyT2^+^ PnC neurons. Data are from *n* = 18 GlyT2^+^ cells responsive to light and *n* = 20 GlyT2^+^ cells unresponsive to the photo-stimulation.

## Data Availability

All data generated or analyzed during this study are included in this published article and its supplementary information files. The datasets used and/or analyzed during the current study are also available from the corresponding author on reasonable request. Details about the GlyT2-Cre mice can be found in Giber et al. [67]. Details about the GlyT2-eGFP mice can be found in Zeilhofer et al*.* [50].

## Abbreviations

AAV-DJ: Adeno-associated virus serotype DJ
Arch3.0: Archaerhodopsin
ASR: Acoustic startle reflex
au: Arbitrary units
BLA: Basolateral amygdaloid nucleus, anterior part
CamKIIα: Calcium/calmodulin-dependent protein kinase II alpha
CeA: Central amygdaloid nucleus
ChAT: Choline acetyltransferase
ChR2: Channelrhodopsin-2
CN: Cochlear nuclei
CSPP: Corticostriatal-pallido-pontine
eGFP: Enhanced green fluorescent protein
EPSC: Excitatory post-synaptic current
EPSP: Excitatory post-synaptic potential
eYFP: Enhanced yellow fluorescent protein
GABA: Gamma-aminobutyric acid
GlyT2: Glycine transporter 2
IC: Inferior colliculus
IPSC: Inhibitory post-synaptic current
IPSP: Inhibitory post-synaptic potential
ISI: Interstimulus interval
MNs: Motor neurons
NpHR3.0: Halorhodopsin
PnC: Caudal pontine reticular nucleus
PPI: Prepulse inhibition
PPTg: Pedunculopontine tegmental nucleus
rlu: Relative light units
SC: Superior colliculus
VGLUT2: Vesicular glutamate transporter 2

## References

[1] Braff DL, Stone C, Callaway E, Geyer M, Glick I, Bali L (1978). Prestimulus effects on human startle reflex in normals and schizophrenics. Psychophysiology..

[2] Swerdlow NR, Braff DL, Geyer MA (1999). Cross-species studies of sensorimotor gating of the startle reflex. Ann N Y Acad Sci..

[3] Perry W, Braff DL (1994). Information-processing deficits and thought disorder in schizophrenia. Am J Psychiatry..

[4] Li L, Du Y, Li N, Wu X, Wu Y (2009). Top-down modulation of prepulse inhibition of the startle reflex in humans and rats. Neurosci Biobehav Rev..

[5] Swerdlow NR, Benbow CH, Zisook S, Geyer MA, Braff DL (1993). A preliminary assessment of sensorimotor gating in patients with obsessive compulsive disorder. Biol Psychiatry..

[6] Hoenig K, Hochrein A, Quednow BB, Maier W, Wagner M (2005). Impaired prepulse inhibition of acoustic startle in obsessive-compulsive disorder. Biol Psychiatry..

[7] Castellanos FX, Fine EJ, Kaysen D, Marsh WL, Rapoport JL, Hallett M (1996). Sensorimotor gating in boys with Tourette’s syndrome and ADHD: preliminary results. Biol Psychiatry..

[8] Grillon C, Morgan CA, Southwick SM, Davis M, Charney DS (1996). Baseline startle amplitude and prepulse inhibition in Vietnam veterans with posttraumatic stress disorder. Psychiatry Res..

[9] Graham FK (1975). The more or less startling effects of weak prestimulation. Psychophysiology..

[10] Muñoz E, Cervera A, Valls-Solé J (2003). Neurophysiological study of facial chorea in patients with Huntington’s disease. Clin Neurophysiol..

[11] Swerdlow NR, Paulsen J, Braff DL, Butters N, Geyer MA, Swenson MR (1995). Impaired prepulse inhibition of acoustic and tactile startle response in patients with Huntington's disease. J Neurol Neurosurg Psychiatry..

[12] Valls-Solé J, Muñoz JE, Valldeoriola F (2004). Abnormalities of prepulse inhibition do not depend on blink reflex excitability: a study in Parkinson’s disease and Huntington’s disease. Clin Neurophysiol..

[13] Morton WA, Laird LK, Crane DF, Partovi N, Frye LH (1994). A prediction model for identifying alcohol withdrawal seizures. Am J Drug Alcohol Abuse..

[14] Pouretemad HR, Thompson PJ, Fenwick PB (1998). Impaired sensorimotor gating in patients with non-epileptic seizures. Epilepsy Res..

[15] Kızıltan ME, Alpaslan BG, Özkara Ç, Uzan M, Gündüz A (2018). Role of mesial temporal lobe structures in sensory processing in humans: a prepulse modulation study in temporal lobe epilepsy. Exp Brain Res..

[16] Ornitz EM, Russell AT, Hanna GL, Gabikian P, Gehricke JG, Song D, Guthrie D (1999). Prepulse inhibition of startle and the neurobiology of primary nocturnal enuresis. Biol Psychiatry..

[17] Kumari V, Soni W, Sharma T (1999). Normalization of information processing deficits in schizophrenia with clozapine. Am J Psychiatry..

[18] Ribeiro BM, do Carmo MR, Freire RS, Rocha NF, Borella VC, de Menezes AT, Monte AS, Gomes PX, de Sousa FC, Vale ML, de Lucena DF, Gama CS, Macêdo D (2013). Evidences for a progressive microglial activation and increase in iNOS expression in rats submitted to a neurodevelopmental model of schizophrenia: reversal by clozapine. Schizophr Res..

[19] Davis M, Gendelman DS, Tischler MD, Gendelman PM (1982). A primary acoustic startle circuit: lesion and stimulation studies. J Neurosci..

[20] Lee Y, López DE, Meloni EG, Davis M (1996). A primary acoustic startle pathway: obligatory role of cochlear root neurons and the nucleus reticularis pontis caudalis. J Neurosci..

[21] Semba K, Fibiger HC (1992). Afferent connections of the laterodorsal and the pedunculopontine tegmental nuclei in the rat: a retro- and antero-grade transport and immunohistochemical study. J Comp Neurol..

[22] Swerdlow NR, Geyer MA (1993). Prepulse inhibition of acoustic startle in rats after lesions of the pedunculopontine tegmental nucleus. Behav Neurosci..

[23] Koch M, Kungel M, Herbert H (1993). Cholinergic neurons in the pedunculopontine tegmental nucleus are involved in the mediation of prepulse inhibition of the acoustic startle response in the rat. Exp Brain Res..

[24] Yeomans JS, Lee J, Yeomans MH, Steidl S, Li L (2006). Midbrain pathways for prepulse inhibition and startle activation in rat. Neuroscience..

[25] Swerdlow NR, Geyer MA, Braff DL (2001). Neural circuit regulation of prepulse inhibition of startle in the rat: current knowledge and future challenges. Psychopharmacology (Berl)..

[26] MacLaren DA, Markovic T, Clark SD (2014). Assessment of sensorimotor gating following selective lesions of cholinergic pedunculopontine neurons. Eur J Neurosci..

[27] Azzopardi E, Louttit AG, DeOliveira C, Laviolette SR, Schmid S (2018). The role of cholinergic midbrain neurons in startle and prepulse inhibition. J Neurosci..

[28] Fulcher N, Azzopardi E, De Oliveira C, Hudson R, Schormans AL, Zaman T (2020). Deciphering midbrain mechanisms underlying prepulse inhibition of startle. Prog Neurobiol..

[29] Bergeron SA, Carrier N, Li GH, Ahn S, Burgess HA (2015). Gsx1 expression defines neurons required for prepulse inhibition. Mol Psychiatry..

[30] Tabor KM, Smith TS, Brown M, Bergeron SA, Briggman KL, Burgess HA (2018). Presynaptic inhibition selectively gates auditory transmission to the brainstem startle circuit. Curr Biol..

[31] Wang HL, Morales M (2009). Pedunculopontine and laterodorsal tegmental nuclei contain distinct populations of cholinergic, glutamatergic and GABAergic neurons in the rat. Eur J Neurosci..

[32] Curtin PC, Preuss T (2015). Glycine and GABAA receptors mediate tonic and phasic inhibitory processes that contribute to prepulse inhibition in the goldfish startle network. Front Neural Circuits..

[33] Ho NF, Li Hui Chong P, Lee DR, Chew QH, Chen G, Sim K (2019). The amygdala in schizophrenia and bipolar disorder: a synthesis of structural MRI, diffusion tensor imaging, and resting-state functional connectivity findings. Harv Rev Psychiatry..

[34] Decker MW, Curzon P, Brioni JD (1995). Influence of separate and combined septal and amygdala lesions on memory, acoustic startle, anxiety, and locomotor activity in rats. Neurobiol. Learn Mem..

[35] Fendt M, Schwienbacher I, Koch M (2000). Amygdaloid N-methyl-D-aspartate and gamma-aminobutyric acid(A) receptors regulate sensorimotor gating in a dopamine-dependent way in rats. Neuroscience..

[36] Koch M, Ebert U (1998). Deficient sensorimotor gating following seizures in amygdala-kindled rats. Biol Psychiatry..

[37] Wan FJ, Swerdlow NR (1997). The basolateral amygdala regulates sensorimotor gating of acoustic startle in the rat. Neurosci..

[38] Howland JG, Hannesson DK, Barnes SJ, Phillips AG (2007). Kindling of basolateral amygdala but not ventral hippocampus or perirhinal cortex disrupts sensorimotor gating in rats. Behav Brain Res..

[39] Kim J, Zhang X, Muralidhar S, LeBlanc SA, Tonegawa S (2017). Basolateral to central amygdala neural circuits for appetitive behaviors. Neuron..

[40] McDonald AJ (1998). Cortical pathways to the mammalian amygdala. Prog Neurobiol..

[41] LeDoux JE, Iwata J, Cicchetti P, Reis DJ (1988). Different projections of the central amygdaloid nucleus mediate autonomic and behavioral correlates of conditioned fear. J Neurosci..

[42] Rosen JB, Hitchcock JM, Sananes CB, Miserendino MJ, Davis M (1991). A direct projection from the central nucleus of the amygdala to the acoustic startle pathway: anterograde and retrograde tracing studies. Behav Neurosci..

[43] Koch M, Ebert U (1993). Enhancement of the acoustic startle response by stimulation of an excitatory pathway from the central amygdala/basal nucleus of Meynert to the pontine reticular formation. Exp Brain Res..

[44] Rosen JB, Davis M (1988). Enhancement of acoustic startle by electrical stimulation of the amygdala. Behav Neurosci..

[45] Hitchcock JM, Davis M (1991). Efferent pathway of the amygdala involved in conditioned fear as measured with the fear-potentiated startle paradigm. Behav Neurosci..

[46] Grillon C, Ameli R, Woods SW, Merikangas K, Davis M (1991). Fear-potentiated startle in humans: effects of anticipatory anxiety on the acoustic blink reflex. Psychophysiology..

[47] Lingenhöhl K, Friauf E (1994). Giant neurons in the rat reticular formation: a sensorimotor interface in the elementary acoustic startle circuit?. J Neurosci..

[48] Tapias-Espinosa C, Río-Álamos C, Sánchez-González A, Oliveras I, Sampedro-Viana D, Castillo-Ruiz MDM (2019). Schizophrenia-like reduced sensorimotor gating in intact inbred and outbred rats is associated with decreased medial prefrontal cortex activity and volume. Neuropsychopharmacology..

[49] Rampon C, Luppi PH, Fort P, Peyron C, Jouvet M (1996). Distribution of glycine-immunoreactive cell bodies and fibers in the rat brain. Neuroscience..

[50] Zeilhofer HU, Studler B, Arabadzisz D, Schweizer C, Ahmadi S, Layh B (2005). Glycinergic neurons expressing enhanced green fluorescent protein in bacterial artificial chromosome transgenic mice. J Comp Neurol.

[51] Geis HR, Schmid S (2011). Glycine inhibits startle-mediating neurons in the caudal pontine reticular formation but is not involved in synaptic depression underlying short-term habituation of startle. Neurosci Res..

[52] Paxinos G, Franklin KBJ (2001). The mouse brain in stereotaxic coordinates.

[53] Rangarajan JR, Vande Velde G, van Gent F, De Vloo P, Dresselaers T, Depypere M (2016). Image-based in vivo assessment of targeting accuracy of stereotactic brain surgery in experimental rodent models. Sci Rep..

[54] McCullough KM, Morrison FG, Hartmann J, Carlezon WA Jr, Ressler KJ. Quantified coexpression analysis of central amygdala subpopulations. eNeuro. 2018;5(1). 10.1523/ENEURO.0010-18.2018. 10.1523/ENEURO.0010-18.2018 PMC5810038 29445764

[55] Fung SJ, Xi M, Zhang J, Torterolo P, Sampogna S, Morales FR, Chase MH (2011). Projection neurons from the central nucleus of the amygdala to the nucleus pontis oralis. J Neurosci Res..

[56] Nathanson JL, Yanagawa Y, Obata K, Callaway EM (2009). Preferential labeling of inhibitory and excitatory cortical neurons by endogenous tropism of adeno-associated virus and lentivirus vectors. Neuroscience..

[57] Hitchcock J, Davis M (1986). Lesions of the amygdala, but not of the cerebellum or red nucleus, block conditioned fear as measured with the potentiated startle paradigm. Behav Neurosci..

[58] Hopkins DA, Holstege G (1978). Amygdaloid projections to the mesencephalon, pons and medulla oblongata in the cat. Exp Brain Res..

[59] Krettek JE, Price JL (1978). Amygdaloid projections to subcortical structures within the basal forebrain and brainstem in the rat and cat. J Comp Neurol..

[60] Price JL, Amaral DG (1981). An autoradiographic study of the projections of the central nucleus of the monkey amygdala. J Neurosci..

[61] Takeuchi Y, Satoda T, Tashiro T, Matsushima R, Uemura-Sumi M (1988). Amygdaloid pathway to the trigeminal motor nucleus via the pontine reticular formation in the rat. Brain Res Bull..

[62] Yasui Y, Tsumori T, Oka T, Yokota S (2004). Amygdaloid axon terminals are in contact with trigeminal premotor neurons in the parvicellular reticular formation of the rat medulla oblongata. Brain Res..

[63] Boissard R, Fort P, Gervasoni D, Barbagli B, Luppi PH (2003). Localization of the GABAergic and non-GABAergic neurons projecting to the sublaterodorsal nucleus and potentially gating paradoxical sleep onset. Eur J Neurosci..

[64] Bosch D, Schmid S (2008). Cholinergic mechanism underlying prepulse inhibition of the startle response in rats. Neurosci..

[65] Kandler K, Herbert H (1991). Auditory projections from the cochlear nucleus to pontine and mesencephalic reticular nuclei in the rat. Brain Res..

[66] Zaman T, De Oliveira C, Smoka M, Narla C, Poulter MO, Schmid S (2017). BK Channels mediate synaptic plasticity underlying habituation in rats. J Neurosci..

[67] Giber K, Diana MA, Plattner V, Dugue GP, Bokor H, Rousseau CV (2015). A subcortical inhibitory signal for behavioral arrest in the thalamus. Nat Neurosci..

[68] Koch M, Friauf E (1995). Glycine receptors in the caudal pontine reticular formation: are they important for the inhibition of the acoustic startle response?. Brain Res..

[69] Waldvogel HJ, Baer K, Eady E, Allen KL, Gilbert RT, Mohler H, Rees MI, Nicholson LFB, Faull RLM (2010). Differential localization of gamma-aminobutyric acid type A and glycine receptor subunits and gephyrin in the human pons, medulla oblongata and uppermost cervical segment of the spinal cord: an immunohistochemical study. J Comp Neurol..

[70] Marsh RR, Hoffman HS, Stitt CL (1976). Eyeblink inhibition by monaural and binaural stimulation: one ear is better than two. Science..

[71] Kumari V, Fannon D, Sumich AL, Sharma T (2007). Startle gating in antipsychotic-naïve first episode schizophrenia patients: one ear is better than two. Psychiatry Res..

[72] Pellet J (1990). Neural organization in the brainstem circuit mediating the primary acoustic head startle: an electrophysiological study in the rat. Physiol Behav..

[73] Frankland PW, Scott BW, Yeomans JS (1995). Axons and synapses mediating electrically evoked startle: collision tests and latency analysis. Brain Res..

[74] Mahn M, Prigge M, Ron S, Levy R, Yizhar O (2016). Biophysical constraints of optogenetic inhibition at presynaptic terminals. Nat Neurosci..

[75] Xi M, Fung SJ, Sampogna S, Chase MH (2011). Excitatory projections from the amygdala to neurons in the nucleus pontis oralis in the rat: an intracellular study. Neuroscience..

[76] Hartley ND, Gaulden AD, Báldi R, Winters ND, Salimando GJ, Rosas-Vidal LE, Jameson A, Winder DG, Patel S (2019). Dynamic remodeling of a basolateral-to-central amygdala glutamatergic circuit across fear states. Nat Neurosci..

[77] Krase W, Koch M, Schnitzler HU (1993). Glutamate antagonists in the reticular formation reduce the acoustic startle response. Neuroreport..

[78] Miserendino MJ, Davis M (1993). NMDA and non-NMDA antagonists infused into the nucleus reticularis pontis caudalis depress the acoustic startle reflex. Brain Res..

[79] Steidl S, Faerman P, Li L, Yeomans JS (2004). Kynurenate in the pontine reticular formation inhibits acoustic and trigeminal nucleus-evoked startle, but not vestibular nucleus-evoked startle. Neuroscience..

[80] Zucker RS, Regehr WG (2002). Short-term synaptic plasticity. Annu Rev Physiol..

[81] Cassell MD, Gray TS, Kiss JZ (1986). Neuronal architecture in the rat central nucleus of the amygdala: a cytological, hodological, and immunocytochemical study. J Comp Neurol..

[82] Gray TS, Magnuson DJ (1987). Neuropeptide neuronal efferents from the bed nucleus of the stria terminalis and central amygdaloid nucleus to the dorsal vagal complex in the rat. J Comp Neurol..

[83] Hur EE, Zaborszky L (2005). Vglut2 afferents to the medial prefrontal and primary somatosensory cortices: a combined retrograde tracing in situ hybridization study [corrected]. J Comp Neurol..

[84] Poulin JF, Castonguay-Lebel Z, Laforest S, Drolet G (2008). Enkephalin co-expression with classic neurotransmitters in the amygdaloid complex of the rat. J Comp Neurol..

[85] Ichikawa T, Ajiki K, Matsuura J, Misawa H (1997). Localization of two cholinergic markers, choline acetyltransferase and vesicular acetylcholine transporter in the central nervous system of the rat: in situ hybridization histochemistry and immunohistochemistry. J Chem Neuroanat..

[86] Swanson LW, Petrovich GD (1998). What is the amygdala?. Trends Neurosci..

[87] Jüngling K, Lange MD, Szkudlarek HJ, Lesting J, Erdmann FS, Doengi M, Kügler S, Pape HC (2015). Increased GABAergic efficacy of central amygdala projections to neuropeptide S neurons in the brainstem during fear memory retrieval. Neuropsychopharmacology..

[88] Saha S, Batten TF, Henderson Z (2000). A GABAergic projection from the central nucleus of the amygdala to the nucleus of the solitary tract: a combined anterograde tracing and electron microscopic immunohistochemical study. Neuroscience..

[89] Benes FM, Berretta S (2001). GABAergic interneurons: implications for understanding schizophrenia and bipolar disorder. Neuropsychopharmacology..

[90] Lewis DA, Volk DW, Hashimoto T (2004). Selective alterations in prefrontal cortical GABA neurotransmission in schizophrenia: a novel target for the treatment of working memory dysfunction. Psychopharmacology..

[91] Forcelli PA, West EA, Murnen AT, Malkova L (2012). Ventral pallidum mediates amygdala-evoked deficits in prepulse inhibition. Behav Neurosci..

[92] Ma J, Leung LS (2011). GABA(B) receptor blockade in the hippocampus affects sensory and sensorimotor gating in Long-Evans rats. Psychopharmacology (Berl)..

[93] Fendt M (1999). Enhancement of prepulse inhibition after blockade of GABA activity within the superior colliculus. Brain Res..

[94] Diederich K, Koch M (2005). Role of the pedunculopontine tegmental nucleus in sensorimotor gating and reward-related behavior in rats. Psychopharmacology (Berl).

[95] Swerdlow NR, Braff DL, Geyer MA (1990). GABAergic projection from nucleus accumbens to ventral pallidum mediates dopamine-induced sensorimotor gating deficits of acoustic startle in rats. Brain Res..

[96] Yeomans JS, Bosch D, Alves N, Daros A, Ure RJ, Schmid S (2010). GABA receptors and prepulse inhibition of acoustic startle in mice and rats. Eur J Neurosci..

